# Supplementary figures and images for: mbkmeans: Fast clustering for single cell data using mini-batch k-means
Source: PLoS Comput Biol. 2021 Jan 26;17(1):e1008625. doi: 10.1371/journal.pcbi.1008625 (PMC7864438; doi:10.1371/journal.pcbi.1008625)

# Memory usage for increasing number of cells

Algorithm    ● k-means    ● ClusterR    ● mbkmeans    ● mbkmeans (HDF5)

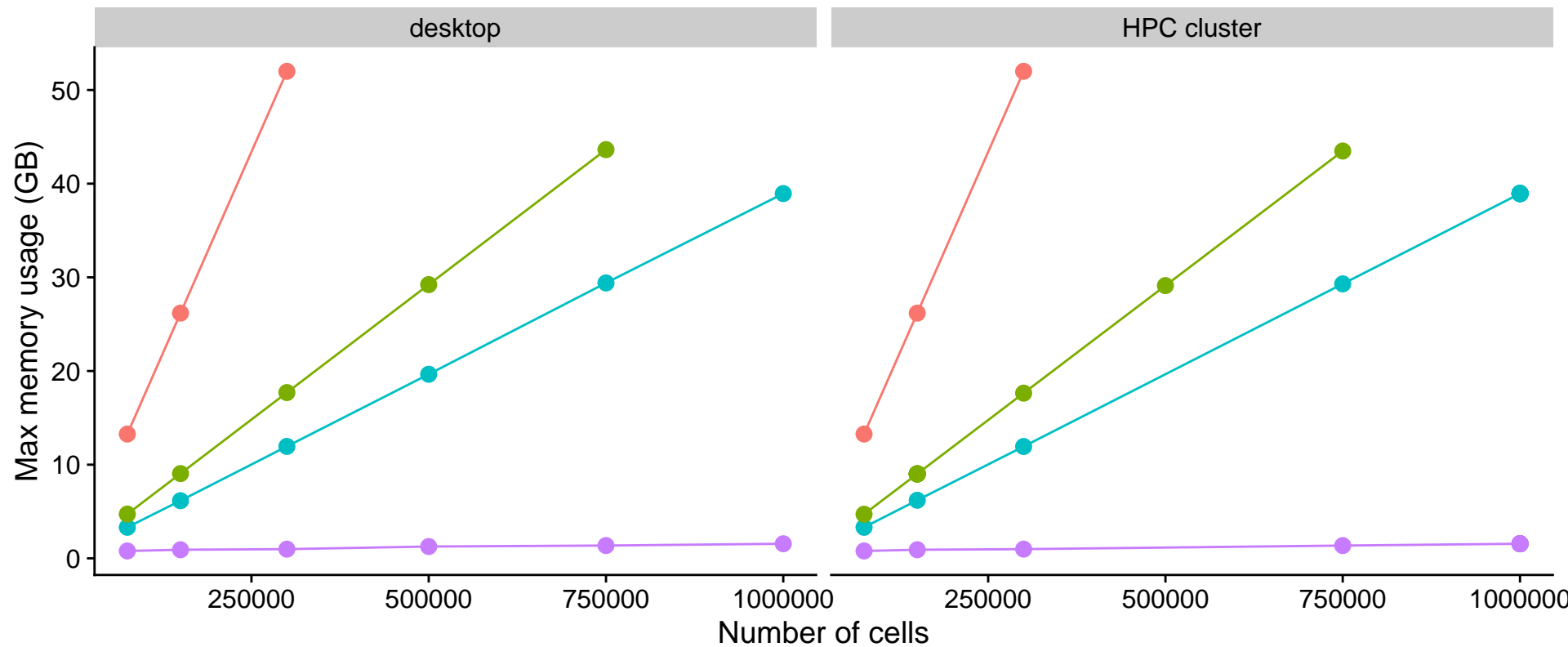

Supplement: S1 Fig — (PDF) [file pcbi.1008625.s001.pdf]

# Elapsed time for increasing number of cells

Algorithm   k-means   ClusterR   mbkmeans   mbkmeans (HDF5)

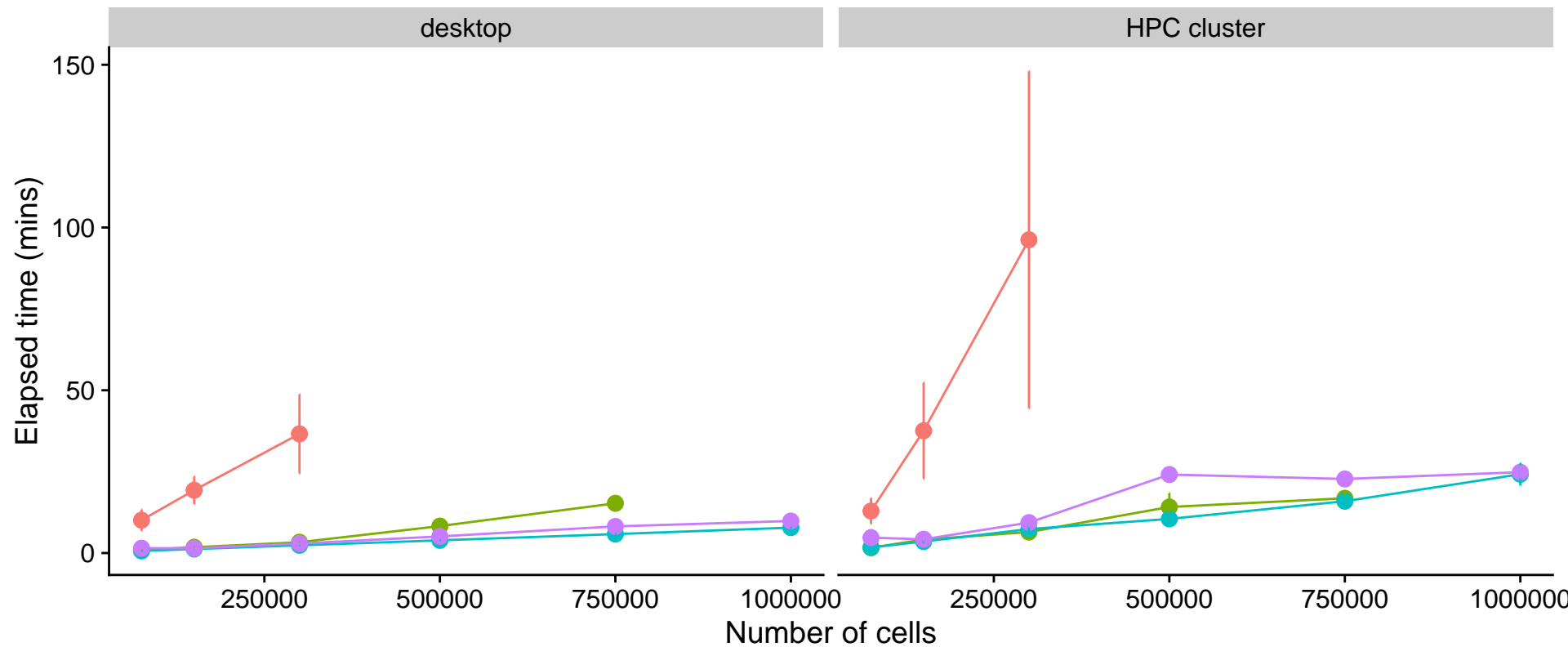

Supplement: S2 Fig — (PDF) [file pcbi.1008625.s002.pdf]

Algorithm    ● k-means    ● mbkmeans    ● mbkmeans (HDF5)

**A**

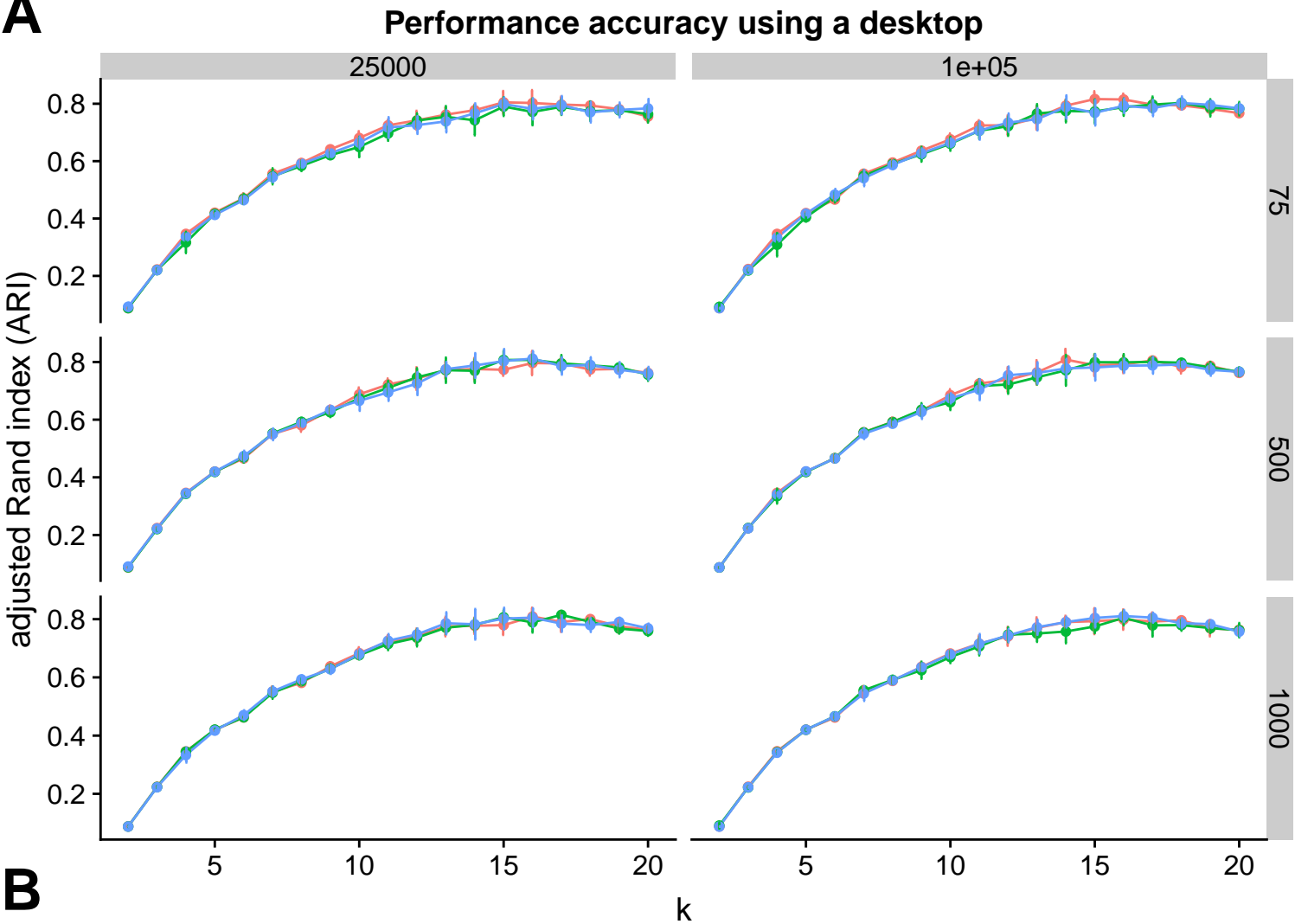

**B**

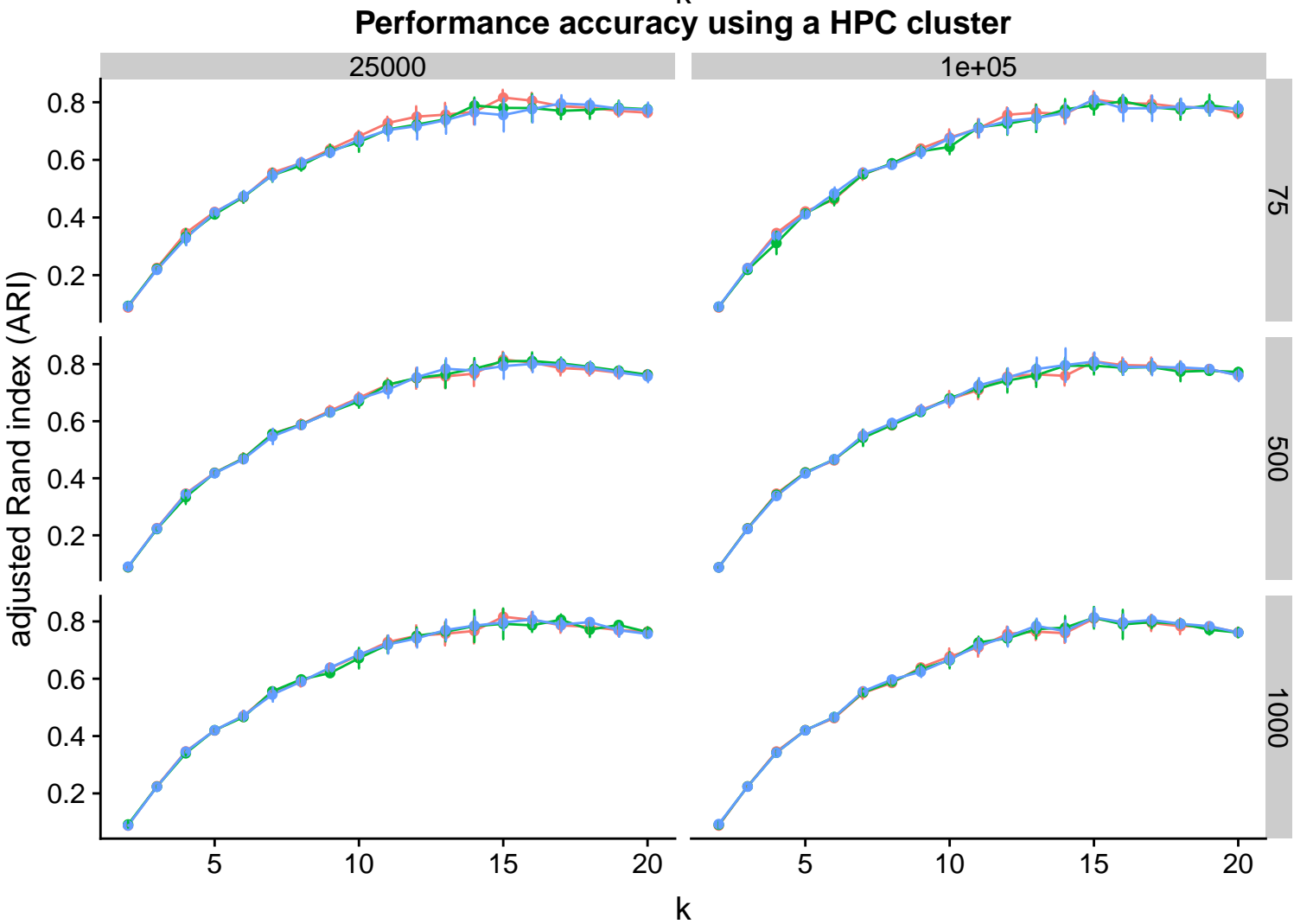

Supplement: S9 Fig — We simulated gene expression data with 15 true centroids for two sizes of datasets (N = 25000, 100000, both using G = 1000 genes) considered three absolute batch sizes of cells (b = 75, 500, 1000) for mbkmeans (both in memory and on-disk using HDF5 files using our desktop configuration). We show the impact of increasing the number of estimated cluster centroids k used in the clustering algorithm (x-axis) on the adjusted Rand index (ARI) performance metric (y-axis). (PDF) [file pcbi.1008625.s009.pdf]

Algorithm    ● k-means    ● mbkmeans    ● mbkmeans (HDF5)

**A**

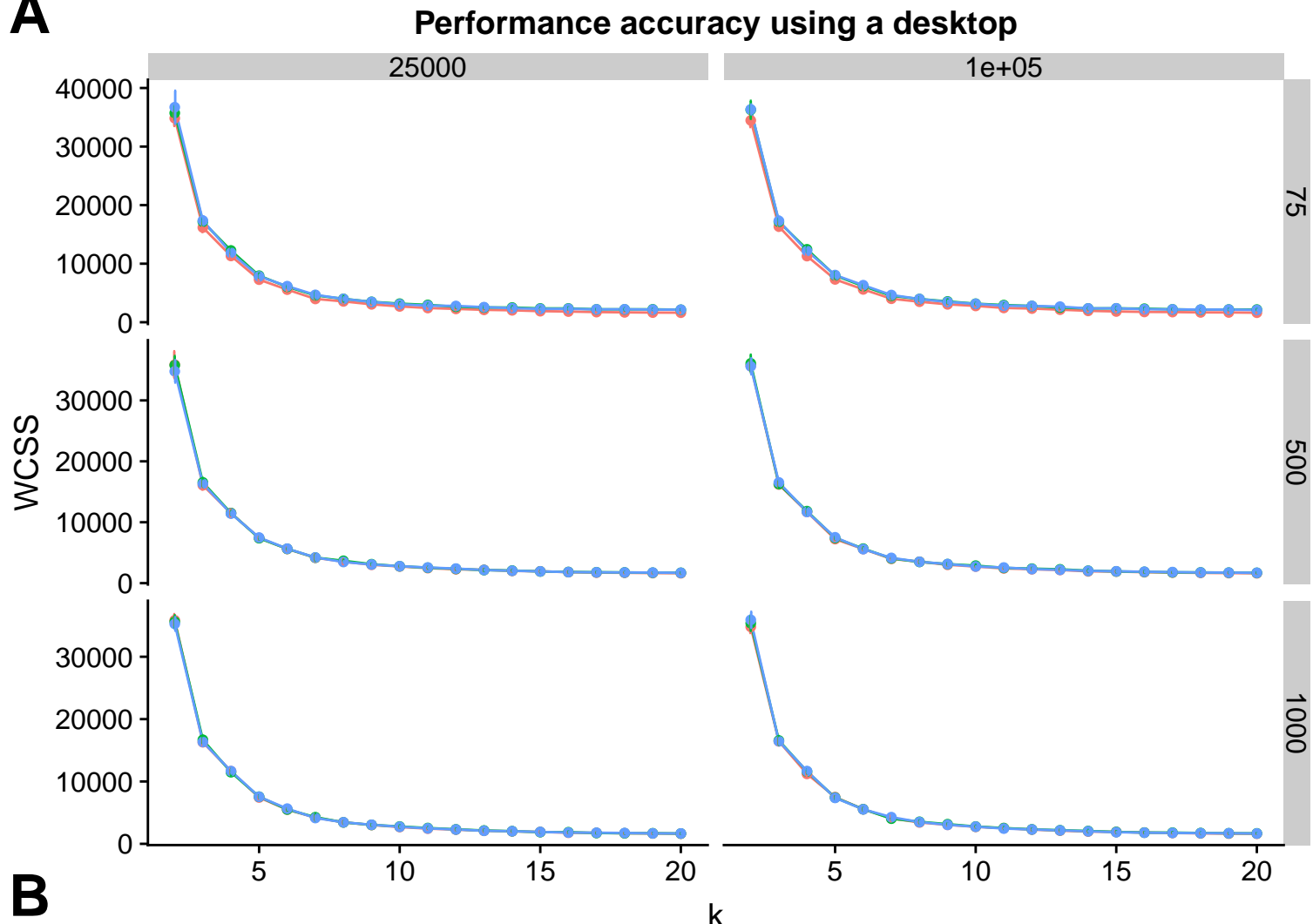

**B**

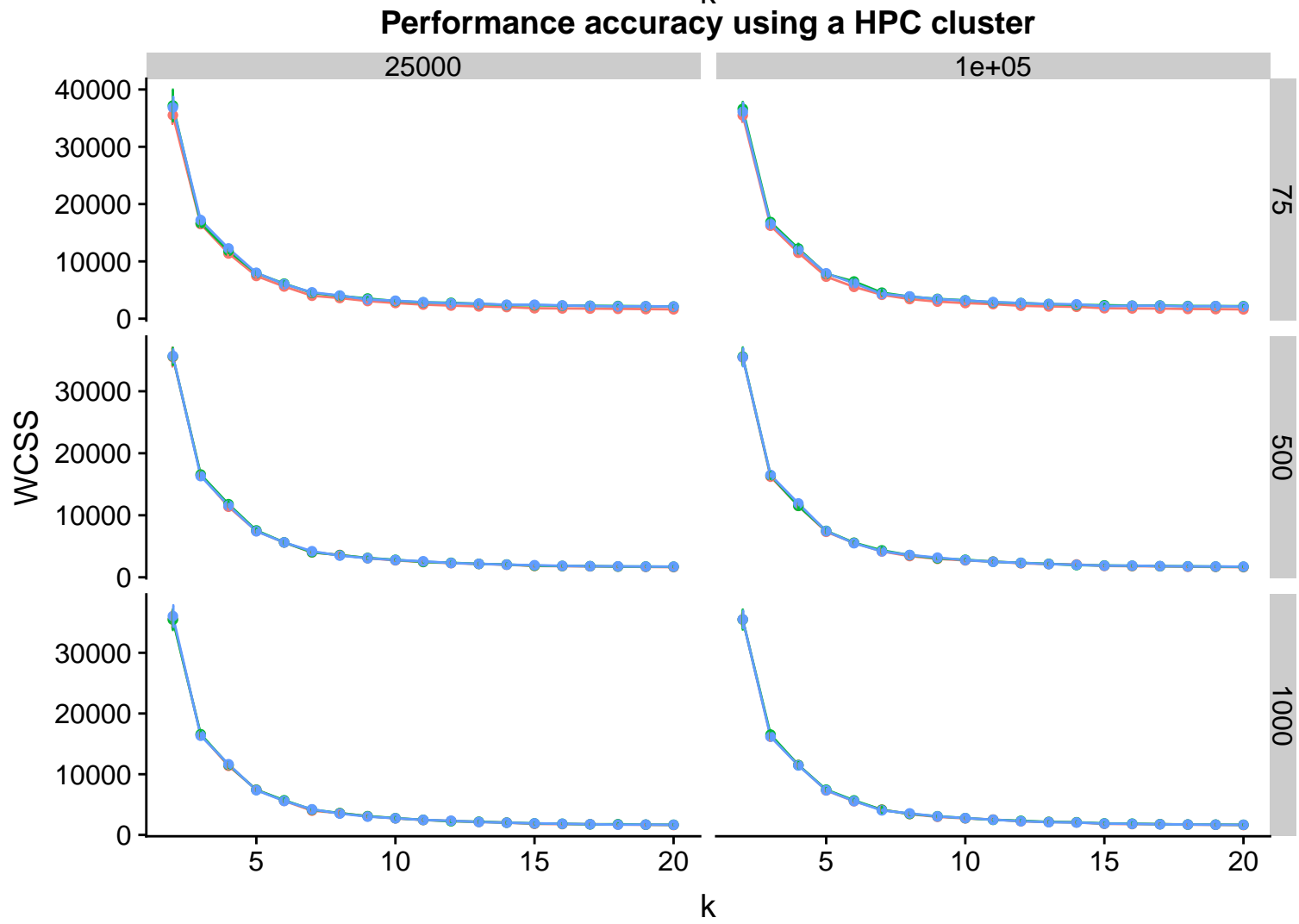

Supplement: S10 Fig — We simulated gene expression data with 15 true centroids for two sizes of datasets (N = 25000, 100000, both using G = 1000 genes) considered three absolute batch sizes of cells (b = 75, 500, 1000) for mbkmeans (both in memory and on-disk using HDF5 files using our desktop configuration). We show the impact of increasing the number of estimated cluster centroids k used in the clustering algorithm (x-axis) on the within clusters sum of squares (WCSS) performance metric (y-axis). (PDF) [file pcbi.1008625.s010.pdf]

# Memory usage for increasing batch size

Algorithm ● mbkmeans ● mbkmeans (HDF5)

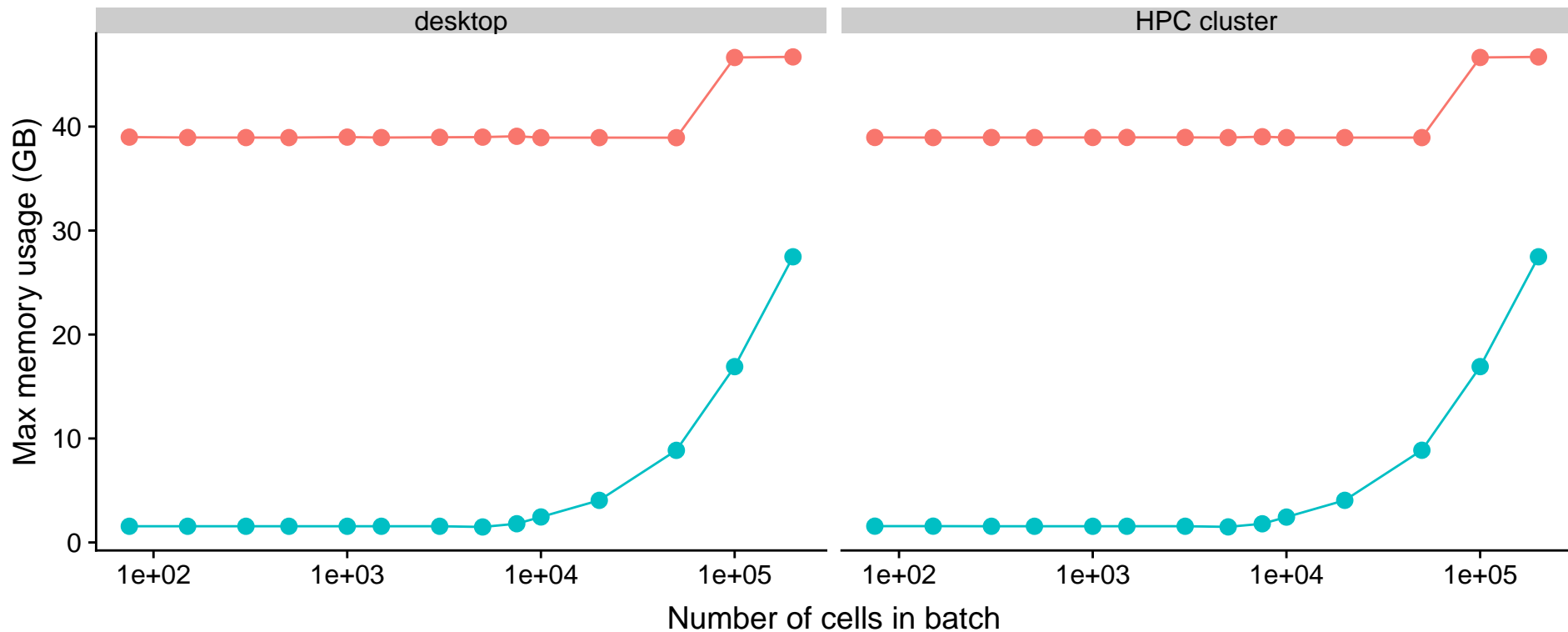

Supplement: S11 Fig — (PDF) [file pcbi.1008625.s011.pdf]

## Elapsed time for increasing batch size

Algorithm    ● mbkmeans    ● mbkmeans (HDF5)

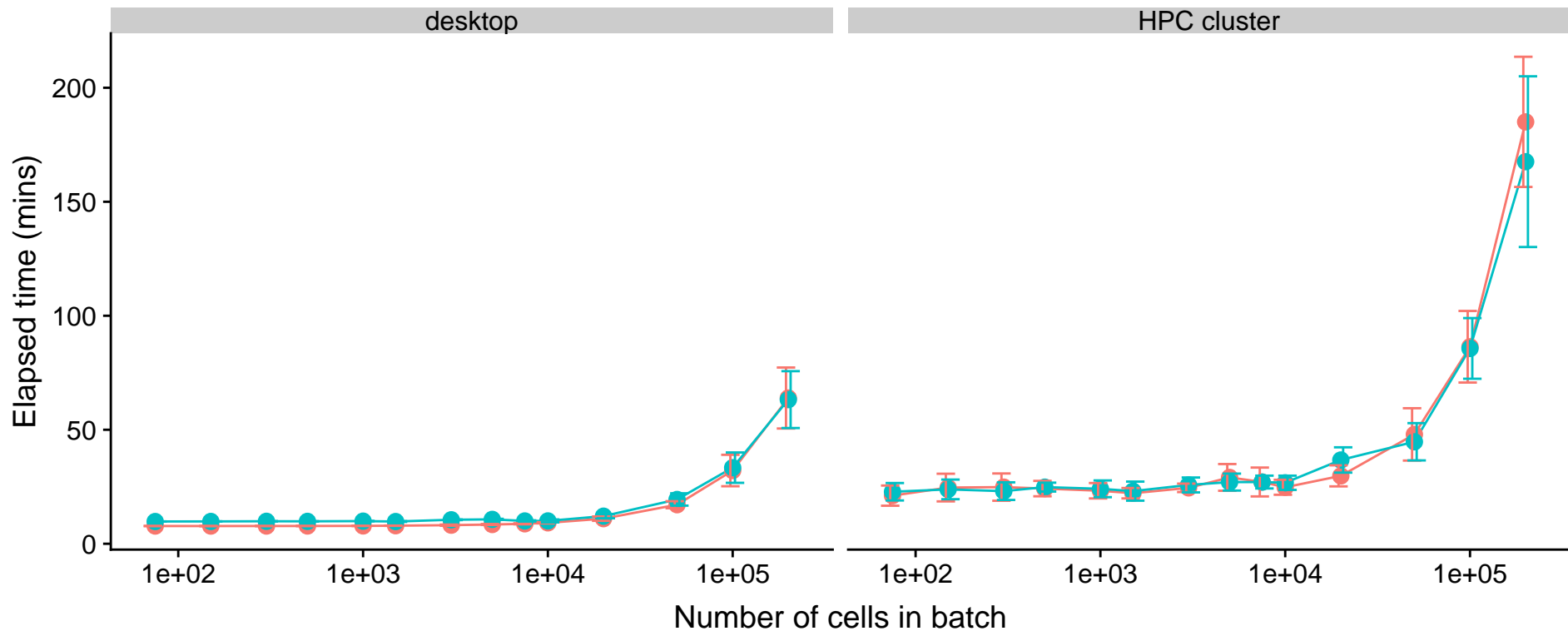

Supplement: S12 Fig — (PDF) [file pcbi.1008625.s012.pdf]

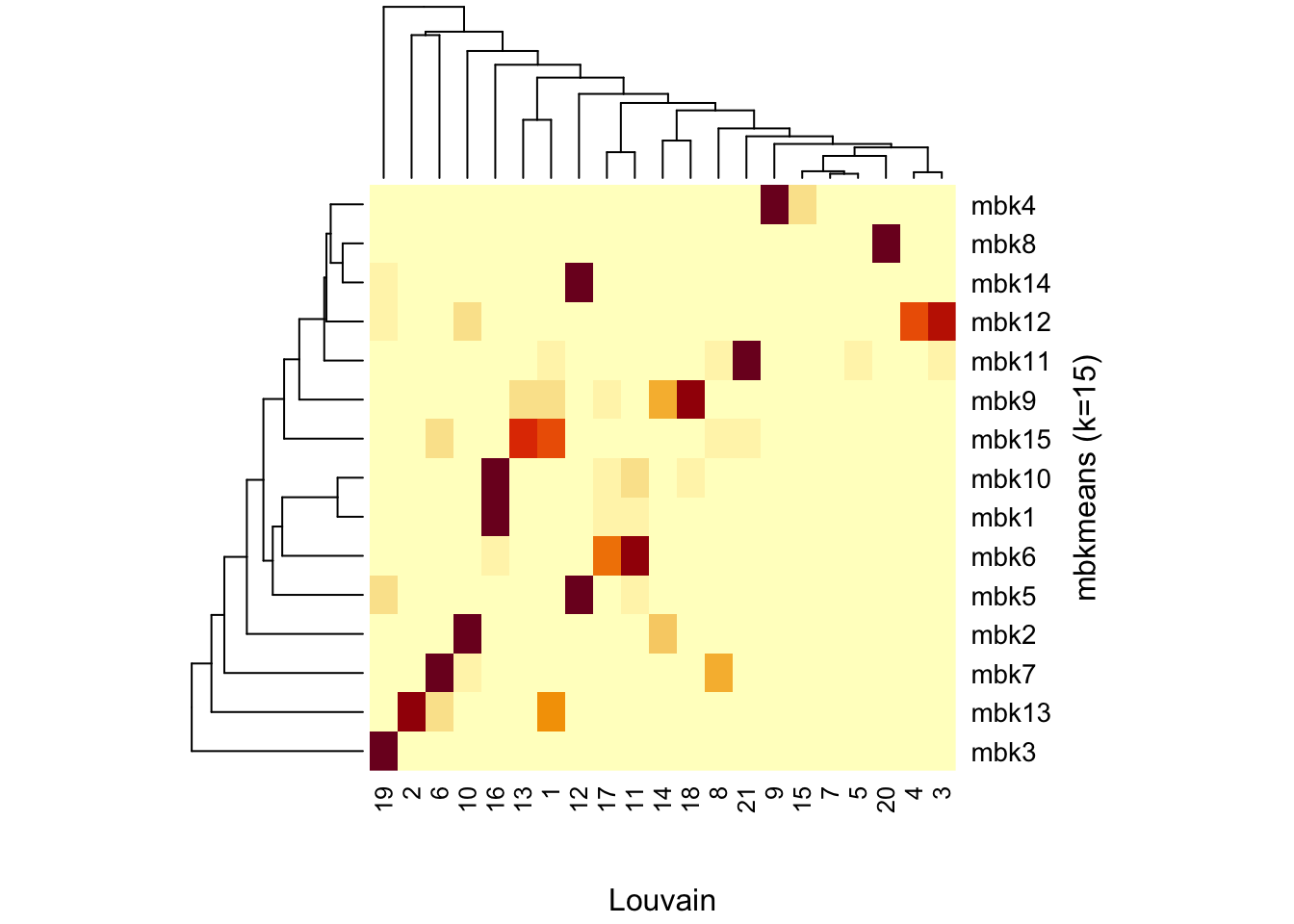

Supplement: S15 Fig — See S1 Text for details on the Louvain clustering. (PNG) [file pcbi.1008625.s015.png]

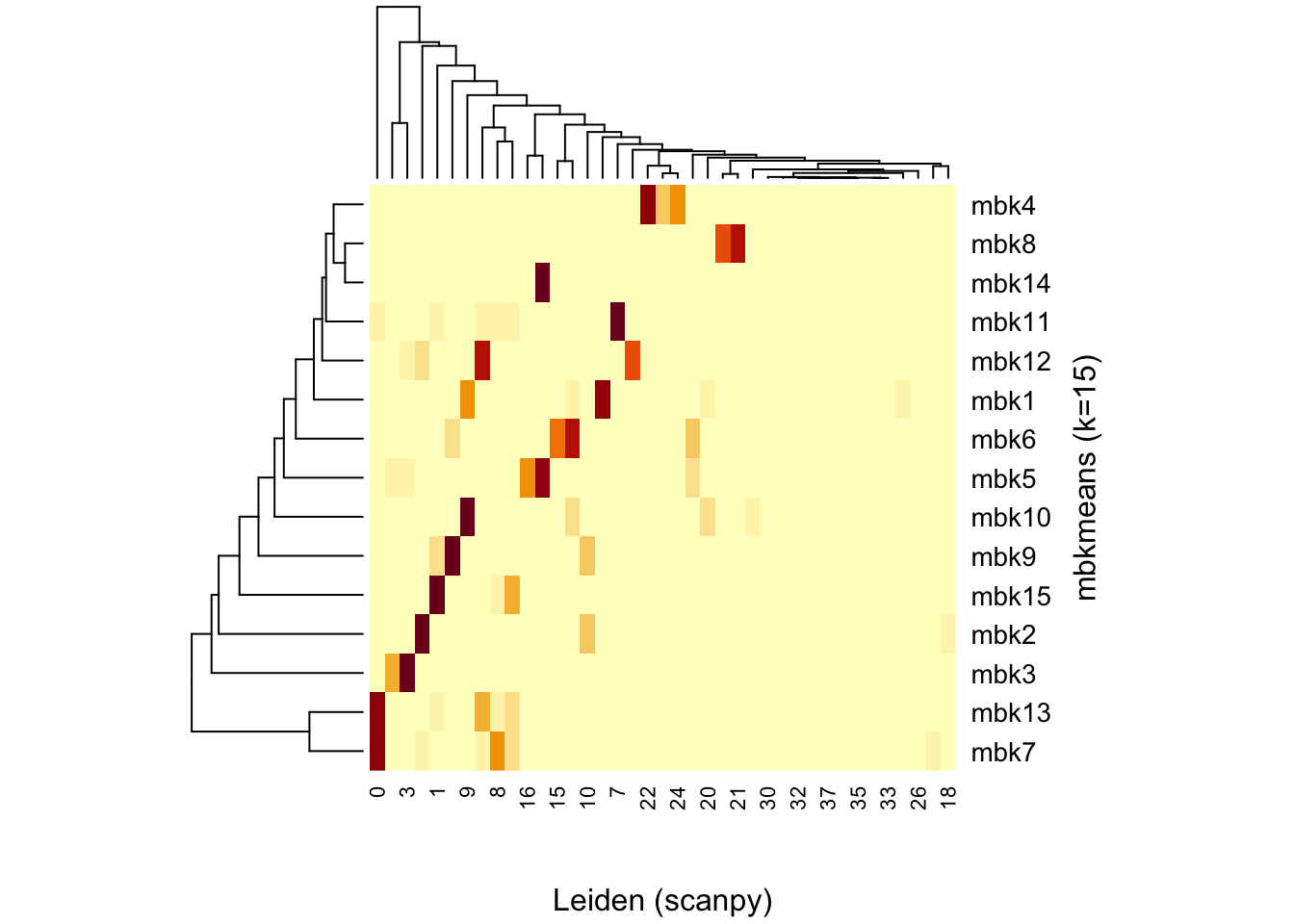

Supplement: S16 Fig — See S1 Text for details on the Leiden clustering. (PNG) [file pcbi.1008625.s016.png]
