# Supplementary material for: mbkmeans: Fast clustering for single cell data using mini-batch k-means
Source: PLoS Comput Biol. 2021 Jan 26;17(1):e1008625. doi: 10.1371/journal.pcbi.1008625 (PMC7864438; doi:10.1371/journal.pcbi.1008625)
Supplement: S6 Fig — (PDF) [file pcbi.1008625.s006.pdf]

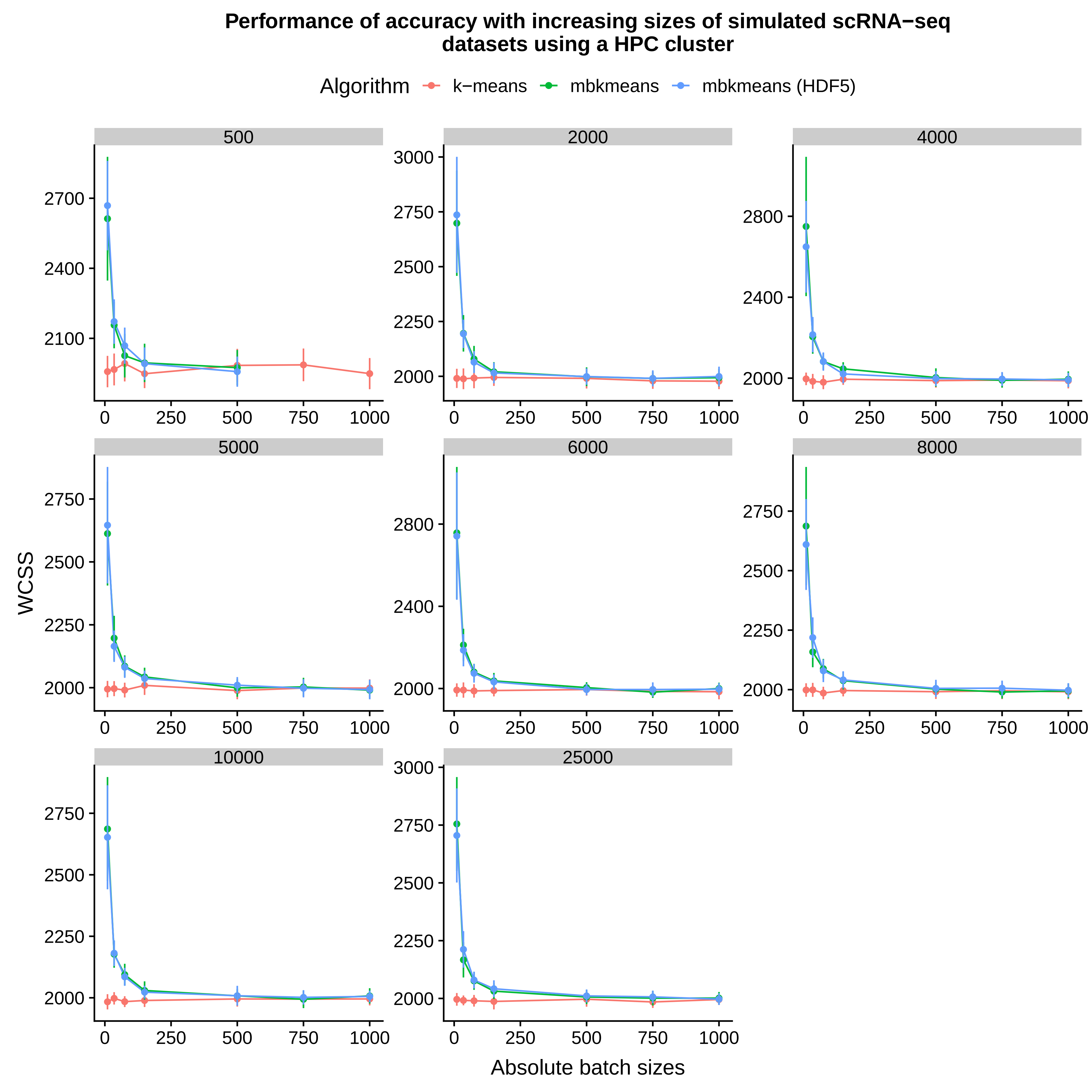

Performance of accuracy with increasing sizes of simulated scRNA−seq datasets using a HPC cluster
Algorithm
k−means
mbkmeans
mbkmeans (HDF5)
500
2000
4000
5000
6000
8000
10000
25000
WCSS
Absolute batch sizes
