# Supplementary material for: mbkmeans: Fast clustering for single cell data using mini-batch k-means
Source: PLoS Comput Biol. 2021 Jan 26;17(1):e1008625. doi: 10.1371/journal.pcbi.1008625 (PMC7864438; doi:10.1371/journal.pcbi.1008625)
Supplement: S7 Fig — (PDF) [file pcbi.1008625.s007.pdf]

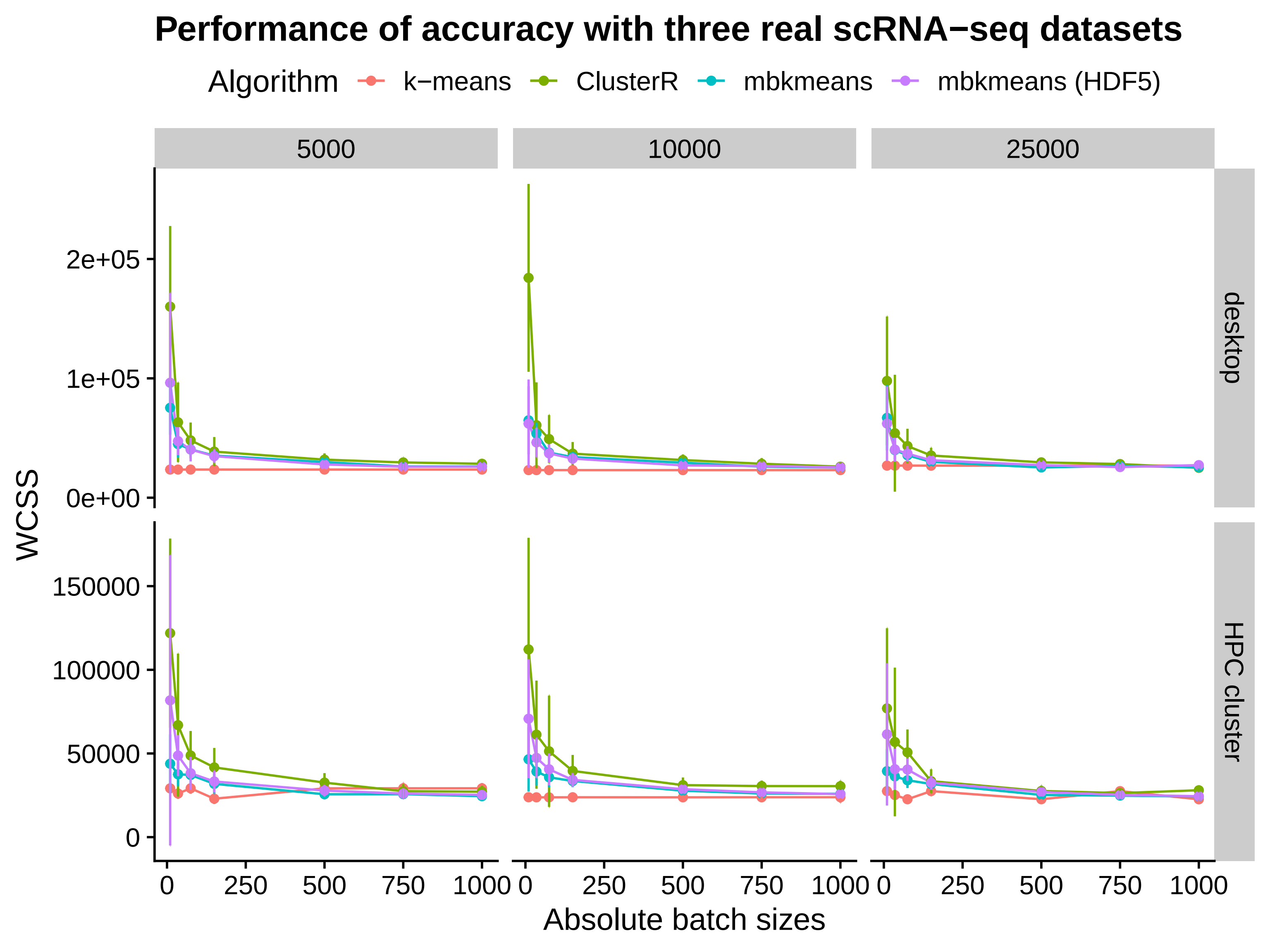

Performance of accuracy with three real scRNA−seq datasets
Algorithm
k−means
ClusterR
mbkmeans
mbkmeans (HDF5)
5000
10000
25000
desktop
HPC cluster
WCSS
2e+05
1e+05
0e+00
150000
100000
50000
0
0
250
500
750
1000
Absolute batch sizes
