# Supplementary material for: mbkmeans: Fast clustering for single cell data using mini-batch k-means
Source: PLoS Comput Biol. 2021 Jan 26;17(1):e1008625. doi: 10.1371/journal.pcbi.1008625 (PMC7864438; doi:10.1371/journal.pcbi.1008625)
Supplement: S8 Fig — (PDF) [file pcbi.1008625.s008.pdf]

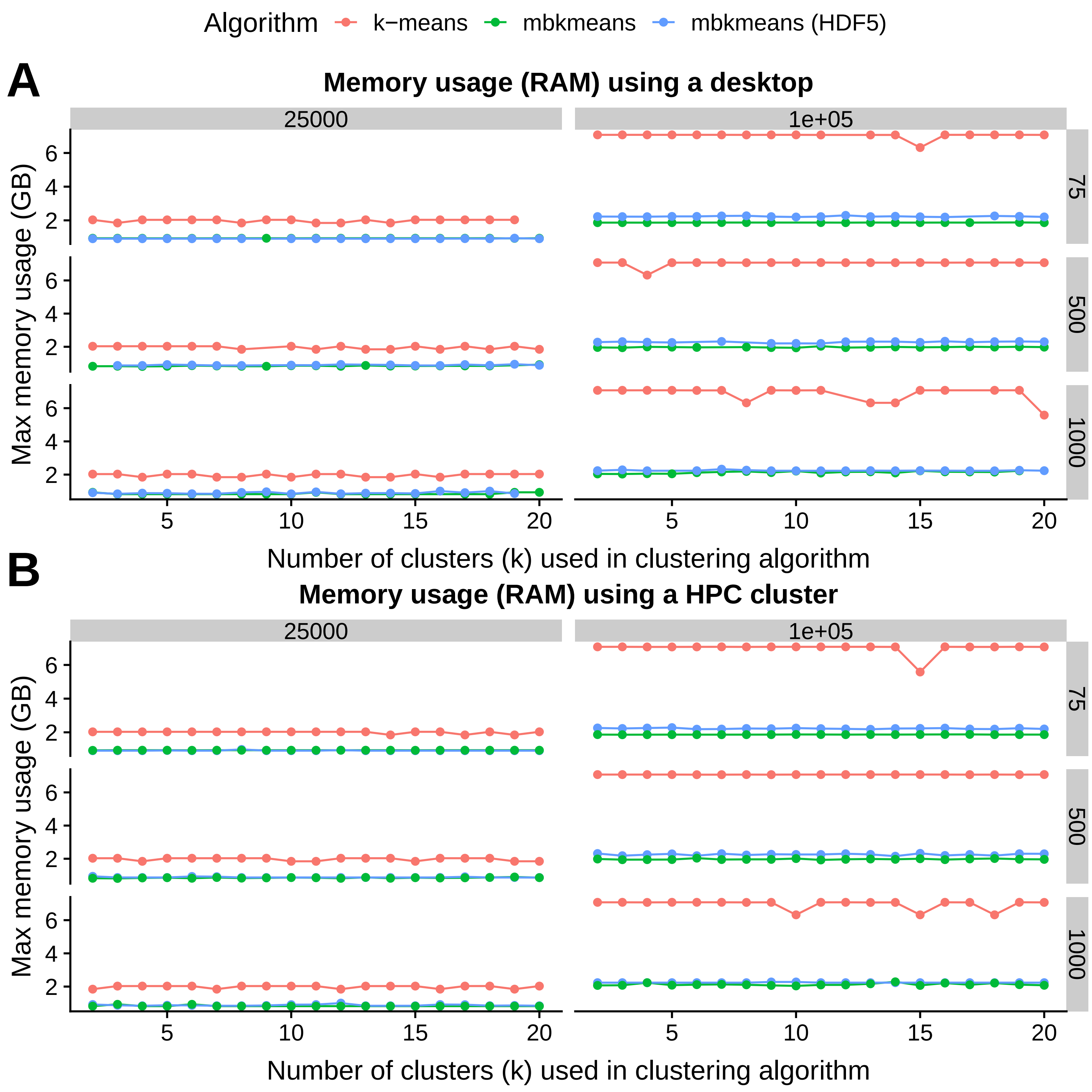
Algorithm
k−means
mbkmeans
mbkmeans (HDF5)
A
Memory usage (RAM) using a desktop
25000
1e+05
75
500
1000
Max memory usage (GB)
Number of clusters (k) used in clustering algorithm
B
Memory usage (RAM) using a HPC cluster
25000
1e+05
75
500
1000
Max memory usage (GB)
Number of clusters (k) used in clustering algorithm
