# Supplementary material for: mbkmeans: Fast clustering for single cell data using mini-batch k-means
Source: PLoS Comput Biol. 2021 Jan 26;17(1):e1008625. doi: 10.1371/journal.pcbi.1008625 (PMC7864438; doi:10.1371/journal.pcbi.1008625)
Supplement: S13 Fig — (PDF) [file pcbi.1008625.s013.pdf]

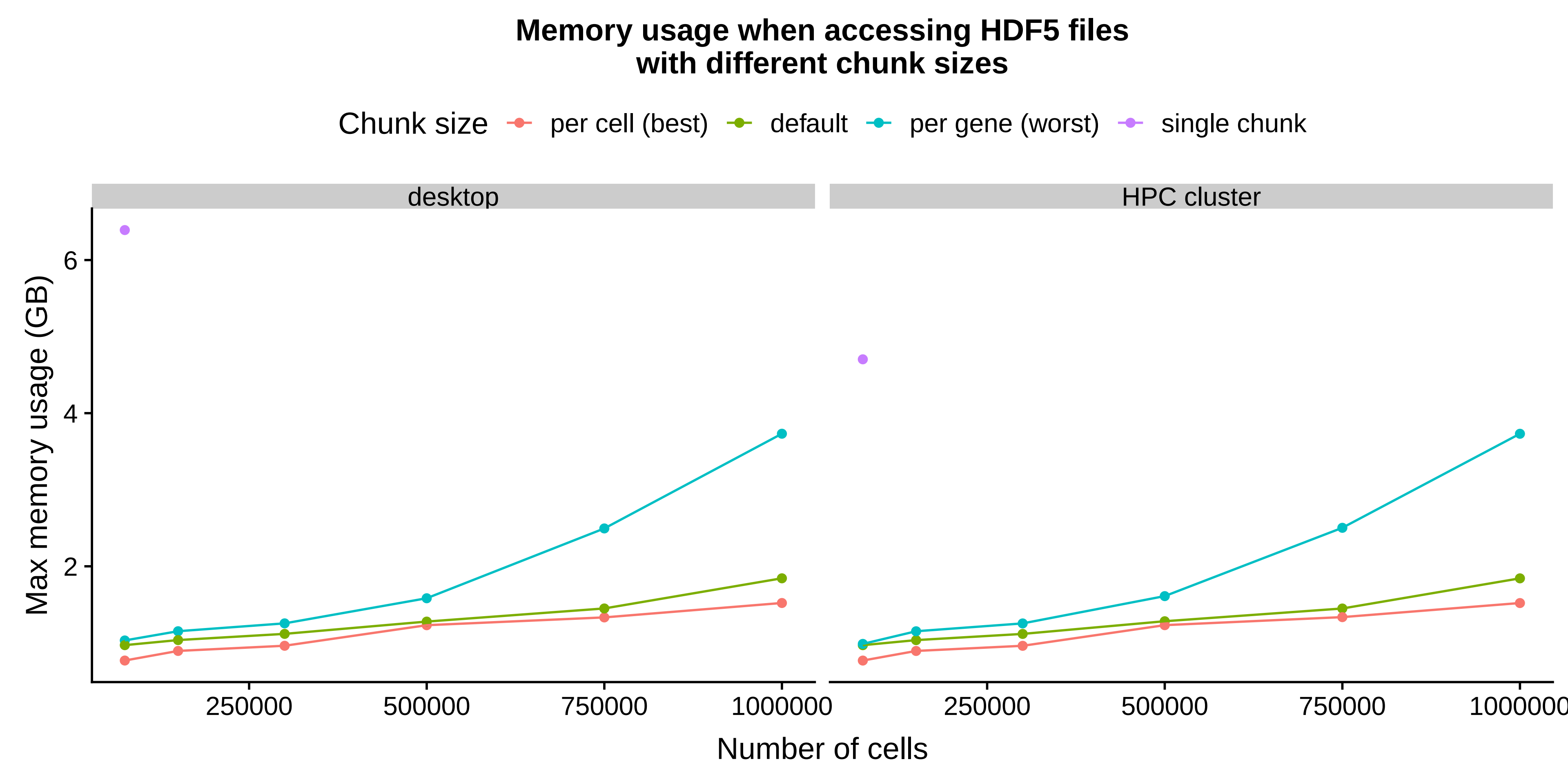
Memory usage when accessing HDF5 files
with different chunk sizes
Chunk size
per cell (best)
default
per gene (worst)
single chunk
desktop
HPC cluster
Max memory usage (GB)
2
4
6
250000
500000
750000
1000000
Number of cells
