# Supplementary material for: mbkmeans: Fast clustering for single cell data using mini-batch k-means
Source: PLoS Comput Biol. 2021 Jan 26;17(1):e1008625. doi: 10.1371/journal.pcbi.1008625 (PMC7864438; doi:10.1371/journal.pcbi.1008625)
Supplement: S14 Fig — (PDF) [file pcbi.1008625.s014.pdf]

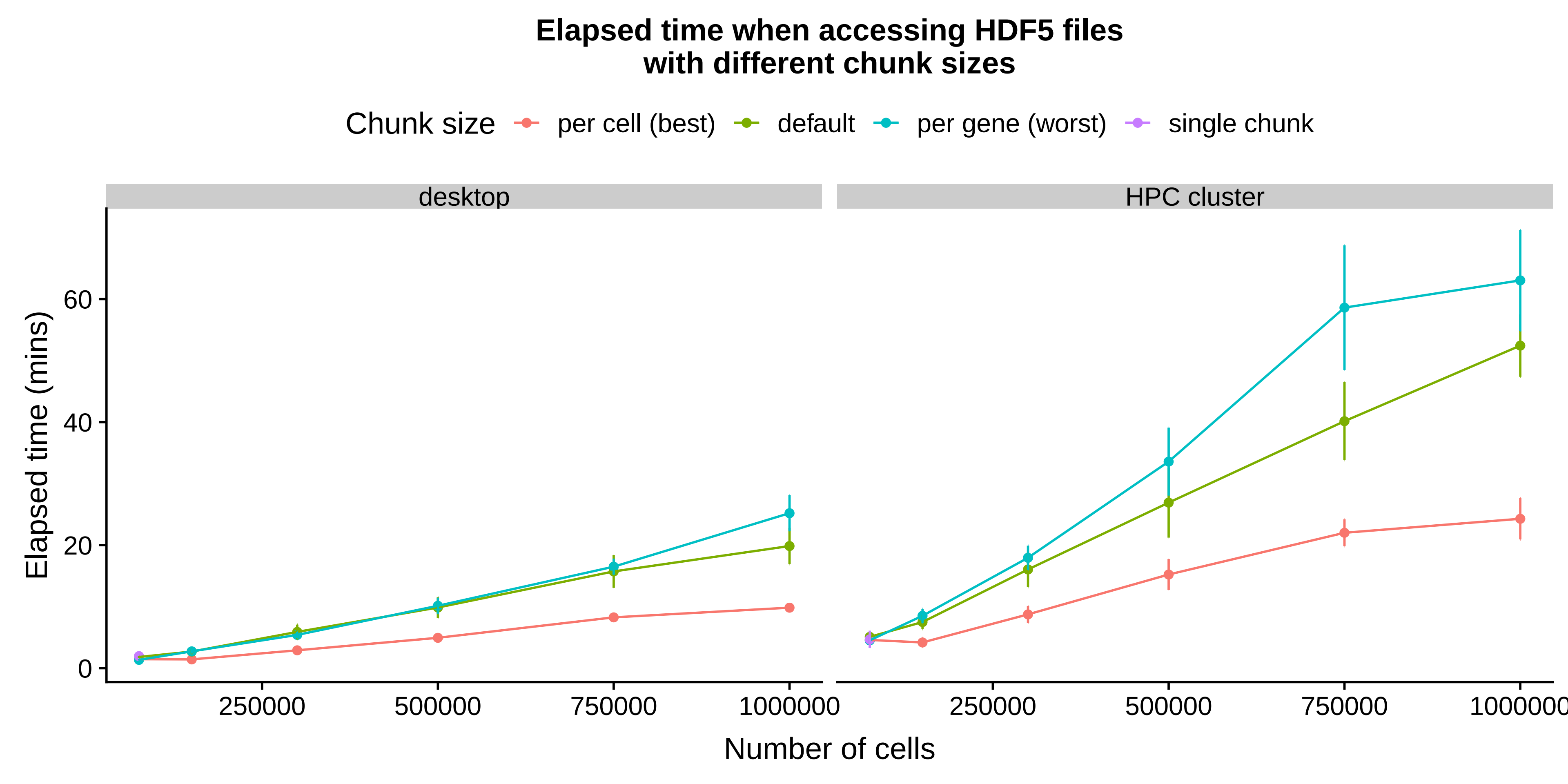

Elapsed time when accessing HDF5 files
with different chunk sizes
Chunk size
per cell (best)
default
per gene (worst)
single chunk
desktop
HPC cluster
Elapsed time (mins)
0
20
40
60
250000
500000
750000
1000000
Number of cells
