# Supplementary material for: mbkmeans: Fast clustering for single cell data using mini-batch k-means
Source: PLoS Comput Biol. 2021 Jan 26;17(1):e1008625. doi: 10.1371/journal.pcbi.1008625 (PMC7864438; doi:10.1371/journal.pcbi.1008625)
Supplement: S1 Table — We report the maximum memory (RAM) used (GB) and averaged elapsed time (minutes) for increasing sizes of datasets with N = 75,000, 150,000, 300,000, 500,000, 750,000, and 1,000,000 observations and 5,000 genes using our desktop computer configuration. The average elapsed time (elapsed_mean) and standard deviation (elapsed_sd) of ten runs is reported in the table. We used k = 15 for both algorithms and used a batch size of b = 500 observations for mbkmeans. (PDF) [file pcbi.1008625.s017.pdf]

S1 Table Performance evaluation for memory-usage and elapsed time as reported in Figure 1. We report the maximum memory (RAM) used (GB) and averaged elapsed time (minutes) for increasing sizes of datasets with N = 75,000, 150,000, 300,000, 500,000, 750,000, and 1,000,000 observations and 5,000 genes using our desktop computer configuration. The average elapsed time (elapsed_mean) and standard deviation (elapsed_sd) of ten runs is reported in the table. We used $k = 15$ for both algorithms and used a batch size of $b = 500$ observations for *mbkmeans*.

Algorithm	ncells	ngenes	batch	memory	elapsed_mean	elapsed_sd
k-means	75000	5000	NA	13.27	10.09	3.25
k-means	150000	5000	NA	26.18	19.29	4.24
k-means	300000	5000	NA	52.00	36.60	12.16
mbkmeans	75000	5000	500	3.32	0.64	0.01
mbkmeans	150000	5000	500	6.16	1.22	0.02
mbkmeans	300000	5000	500	11.95	2.37	0.01
mbkmeans	500000	5000	500	19.65	3.90	0.03
mbkmeans	750000	5000	500	29.40	5.81	0.03
mbkmeans	1000000	5000	500	38.94	7.80	0.04
mbkmeans (HDF5)	75000	5000	500	0.79	1.48	0.04
mbkmeans (HDF5)	150000	5000	500	0.92	1.44	0.03
mbkmeans (HDF5)	300000	5000	500	0.98	2.90	0.03
mbkmeans (HDF5)	500000	5000	500	1.26	5.10	0.30
mbkmeans (HDF5)	750000	5000	500	1.37	8.17	0.06
mbkmeans (HDF5)	1000000	5000	500	1.56	9.83	0.03
