# Supplementary material for: mbkmeans: Fast clustering for single cell data using mini-batch k-means
Source: PLoS Comput Biol. 2021 Jan 26;17(1):e1008625. doi: 10.1371/journal.pcbi.1008625 (PMC7864438; doi:10.1371/journal.pcbi.1008625)
Supplement: S2 Table — We report the adjusted Rand index (ARI) and within-cluster sum of squares (WCSS) averaged across 50 replicates for increasing sizes of datasets with N = 5,000, 10,000, and 25,000 observations and increasing batch sizes b = 10, 35, 75, 150, 500, 750, 1,000 using our desktop computer configuration. The average ARI for simulated data (ari_sim_mean) and standard deviation (ari_sim_sd), average WCSS for simulated data (wcss_sim_mean) and standard deviation (wcss_sim_sd), average WCSS for real scRNA-seq data (ari_real_mean) and standard deviation (ari_real_sd), is reported in the table. We used k = 3 for simulated data and k = 15 for real scRNA-seq data for all algorithms. (PDF) [file pcbi.1008625.s018.pdf]

S2 Table Performance evaluation for accuracy as reported in Figure 2. We report the adjusted Rand index (ARI) and within-cluster sum of squares (WCSS) averaged across 50 replicates for increasing sizes of datasets with N = 5,000, 10,000, and 25,000 observations and increasing batch sizes b = 10, 35, 75, 150, 500, 750, 1,000 using our desktop computer configuration. The average ARI for simulated data (ari_sim_mean) and standard deviation (ari_sim_sd), average WCSS for simulated data (wcss_sim_mean) and standard deviation (wcss_sim_sd), average WCSS for real scRNA-seq data (ari_real_mean) and standard deviation (ari_real_sd), is reported in the table. We used $k = 3$ for simulated data and $k = 15$ for real scRNA-seq data for all algorithms.

Algorithm	ncells	batch	ari_sim_mean	ari_sim_sd	wcss_sim_mean	wcss_sim_sd	wcss_real_mean	wcss_real_sd
k-means	5000	10	0.99	0.00	1991.97	33.95	23602.62	2763.31
k-means	5000	35	0.99	0.00	1994.01	43.30	23603.06	2801.22
k-means	5000	75	0.99	0.00	1988.56	38.73	23610.52	2963.92
k-means	5000	150	0.99	0.00	1986.49	32.85	23613.23	2847.70
k-means	5000	500	0.99	0.00	1993.48	33.45	23598.67	2859.05
k-means	5000	750	0.99	0.00	1986.46	30.49	23597.09	2588.85
k-means	5000	1000	0.99	0.00	1991.07	26.37	23597.66	2809.38
k-means	10000	10	0.99	0.00	1995.62	31.83	23134.54	2675.89
k-means	10000	35	0.99	0.00	1988.50	25.67	23134.54	2918.71
k-means	10000	75	0.99	0.00	1998.80	35.01	23126.82	2718.44
k-means	10000	150	0.99	0.00	1989.61	28.83	23072.22	2748.87
k-means	10000	500	0.99	0.00	1984.55	30.21	23126.74	2757.79
k-means	10000	750	0.99	0.00	1991.72	27.54	23072.42	2698.04
k-means	10000	1000	0.99	0.00	1992.96	27.05	23130.62	2830.83
k-means	25000	10	0.99	0.00	1992.59	27.67	26848.05	2457.03
k-means	25000	35	0.99	0.00	1988.43	28.06	26847.19	2405.62
k-means	25000	75	0.99	0.00	1998.43	27.75	26886.25	1915.76
k-means	25000	150	0.99	0.00	1988.22	29.89	26802.16	2502.97
k-means	25000	500	0.99	0.00	1994.71	26.17	26847.25	2400.46
k-means	25000	750	0.99	0.00	1994.47	25.20	26886.50	2158.91
k-means	25000	1000	0.99	0.00	1989.88	27.91	26848.05	2363.99
mbkmeans	5000	10	0.98	0.01	2787.16	268.53	75926.52	36810.37
mbkmeans	5000	35	0.99	0.00	2190.10	56.82	47257.80	13934.28
mbkmeans	5000	75	0.99	0.00	2076.65	52.05	41642.46	8370.06
mbkmeans	5000	150	0.99	0.00	2031.93	39.00	36584.62	6113.03
mbkmeans	5000	500	0.99	0.00	2005.88	34.43	30958.60	3244.96
mbkmeans	5000	750	0.99	0.00	2002.36	35.44	26249.06	3126.91
mbkmeans	5000	1000	0.99	0.00	1994.01	35.98	26275.76	3167.85
mbkmeans	10000	10	0.98	0.02	2731.52	231.80	66775.23	57543.71
mbkmeans	10000	35	0.99	0.00	2191.87	56.40	54591.46	11713.38
mbkmeans	10000	75	0.99	0.00	2072.60	40.29	38495.49	8133.57
mbkmeans	10000	150	0.99	0.00	2036.44	33.41	34035.43	4781.71
mbkmeans	10000	500	0.99	0.00	2006.83	31.00	30586.92	3621.35
mbkmeans	10000	750	0.99	0.00	2009.23	29.13	26004.16	2621.28
mbkmeans	10000	1000	0.99	0.00	2005.89	37.91	25230.06	2222.69
mbkmeans	25000	10	0.98	0.01	2681.96	217.64	73934.80	37316.23
mbkmeans	25000	35	0.99	0.00	2184.49	68.02	40138.51	8281.45
mbkmeans	25000	75	0.99	0.00	2075.56	34.99	35590.41	5308.87
mbkmeans	25000	150	0.99	0.00	2048.23	29.86	30315.25	3654.87
mbkmeans	25000	500	0.99	0.00	2005.89	27.31	25393.64	2400.27
mbkmeans	25000	750	0.99	0.00	2003.31	28.53	27233.51	2648.95
mbkmeans	25000	1000	0.99	0.00	1998.27	28.00	25523.37	2613.95
mbkmeans (HDF5)	5000	10	0.97	0.06	2700.63	279.54	92209.53	58576.45
mbkmeans (HDF5)	5000	35	0.99	0.00	2192.91	78.10	50082.42	13021.59
mbkmeans (HDF5)	5000	75	0.99	0.00	2077.28	48.34	40336.12	9929.52
mbkmeans (HDF5)	5000	150	0.99	0.00	2036.51	38.06	35382.73	6623.13
mbkmeans (HDF5)	5000	500	0.99	0.00	2008.06	31.28	28177.16	3663.00
mbkmeans (HDF5)	5000	750	0.99	0.00	2003.05	37.56	26082.47	2652.00
mbkmeans (HDF5)	5000	1000	0.99	0.00	2005.52	32.73	26161.00	3154.11
mbkmeans (HDF5)	10000	10	0.98	0.01	2677.68	267.91	62060.41	36992.37
mbkmeans (HDF5)	10000	35	0.99	0.00	2202.86	86.00	46748.12	14494.10
mbkmeans (HDF5)	10000	75	0.99	0.00	2089.67	46.66	36736.58	6796.66
mbkmeans (HDF5)	10000	150	0.99	0.00	2029.64	28.51	32702.95	4389.67
mbkmeans (HDF5)	10000	500	0.99	0.00	2000.40	30.03	26901.48	2629.06
mbkmeans (HDF5)	10000	750	0.99	0.00	1998.41	21.71	26291.87	2835.79
mbkmeans (HDF5)	10000	1000	0.99	0.00	1998.20	37.25	25382.89	2874.41
mbkmeans (HDF5)	25000	10	0.98	0.02	2700.20	236.98	62142.26	31252.64
mbkmeans (HDF5)	25000	35	0.99	0.00	2182.47	61.61	40465.15	14752.19
mbkmeans (HDF5)	25000	75	0.99	0.00	2085.87	43.29	36327.36	5801.33
mbkmeans (HDF5)	25000	150	0.99	0.00	2038.02	26.92	31323.11	3406.76
mbkmeans (HDF5)	25000	500	0.99	0.00	2000.49	19.20	27258.32	2672.19
mbkmeans (HDF5)	25000	750	0.99	0.00	1993.18	26.25	25686.95	2587.39
mbkmeans (HDF5)	25000	1000	0.99	0.00	1996.80	32.75	26705.52	2546.07
