# Supplementary material for: mbkmeans: Fast clustering for single cell data using mini-batch k-means
Source: PLoS Comput Biol. 2021 Jan 26;17(1):e1008625. doi: 10.1371/journal.pcbi.1008625 (PMC7864438; doi:10.1371/journal.pcbi.1008625)
Supplement: S3 Table — We report the maximum memory (RAM) used (GB) and averaged elapsed time (minutes) for increasing batch sizes with b = 75, 150, 300, 500, 1,000, 1,500, 3,000, 5,000, 7,500, 10,000, 20,000, 50,000, 100,000, 200,000 with a dataset of size N = 1,000,000 observations and 5,000 genes using our desktop computer configuration. The average (elapsed_mean) and standard deviation (elapsed_sd) of ten runs is reported in the table. We used k = 15 for the number of centroids in mbkmeans. (PDF) [file pcbi.1008625.s019.pdf]

S3 Table Performance evaluation for memory-usage and elapsed time reported in Figure 3. We report the maximum memory (RAM) used (GB) and averaged elapsed time (minutes) for increasing batch sizes with b = 75, 150, 300, 500, 1,000, 1,500, 3,000, 5,000, 7,500, 10,000, 20,000, 50,000, 100,000, 200,000 with a dataset of size N = 1,000,000 observations and 5,000 genes using our desktop computer configuration. The average (elapsed_mean) and standard deviation (elapsed_sd) of ten runs is reported in the table. We used $k = 15$ for the number of centroids in *mbkmeans*.

Algorithm	ncells	ngenes	batch	memory	elapsed_mean	elapsed_sd
mbkmeans	1e+06	5000	75	38.99	7.78	0.02
mbkmeans	1e+06	5000	150	38.95	7.77	0.03
mbkmeans	1e+06	5000	300	38.95	7.78	0.02
mbkmeans	1e+06	5000	500	38.94	7.80	0.04
mbkmeans	1e+06	5000	1000	38.99	7.83	0.02
mbkmeans	1e+06	5000	1500	38.95	7.91	0.07
mbkmeans	1e+06	5000	3000	38.97	8.16	0.26
mbkmeans	1e+06	5000	5000	38.99	8.45	0.21
mbkmeans	1e+06	5000	7500	39.07	8.76	0.23
mbkmeans	1e+06	5000	10000	38.94	9.18	0.26
mbkmeans	1e+06	5000	20000	38.94	11.01	0.95
mbkmeans	1e+06	5000	50000	38.94	17.12	1.54
mbkmeans	1e+06	5000	100000	46.65	32.12	6.90
mbkmeans	1e+06	5000	200000	46.71	63.92	13.38
mbkmeans (HDF5)	1e+06	5000	75	1.56	9.77	0.04
mbkmeans (HDF5)	1e+06	5000	150	1.56	9.79	0.04
mbkmeans (HDF5)	1e+06	5000	300	1.56	9.86	0.06
mbkmeans (HDF5)	1e+06	5000	500	1.56	9.83	0.03
mbkmeans (HDF5)	1e+06	5000	1000	1.56	9.93	0.05
mbkmeans (HDF5)	1e+06	5000	1500	1.56	9.75	0.16
mbkmeans (HDF5)	1e+06	5000	3000	1.56	10.52	0.14
mbkmeans (HDF5)	1e+06	5000	5000	1.49	10.70	0.24
mbkmeans (HDF5)	1e+06	5000	7500	1.80	10.01	0.26
mbkmeans (HDF5)	1e+06	5000	10000	2.45	10.02	0.64
mbkmeans (HDF5)	1e+06	5000	20000	4.05	12.11	0.94
mbkmeans (HDF5)	1e+06	5000	50000	8.86	19.43	2.70
mbkmeans (HDF5)	1e+06	5000	100000	16.91	33.38	6.66
mbkmeans (HDF5)	1e+06	5000	200000	27.48	63.23	12.47
