# Supplementary material for: mbkmeans: Fast clustering for single cell data using mini-batch k-means
Source: PLoS Comput Biol. 2021 Jan 26;17(1):e1008625. doi: 10.1371/journal.pcbi.1008625 (PMC7864438; doi:10.1371/journal.pcbi.1008625)
Supplement: S4 Table — We report the maximum memory (RAM) used (GB) and averaged elapsed time (minutes) for increasing sizes of datasets with N = 75,000, 150,000, 300,000, 500,000, 750,000, and 1,000,000 observations and 5,000 genes using our desktop computer configuration. The average elapsed time (elapsed_mean) and standard deviation (elapsed_sd) of ten runs is reported in the table. The single chunk was only able to run for the smallest dataset size (N = 75,000). We used k = 15 and used a batch size of b = 500 observations. (PDF) [file pcbi.1008625.s020.pdf]

S4 Table Performance evaluation for memory-usage and elapsed time as reported in Figure 4. We report the maximum memory (RAM) used (GB) and averaged elapsed time (minutes) for increasing sizes of datasets with N = 75,000, 150,000, 300,000, 500,000, 750,000, and 1,000,000 observations and 5,000 genes using our desktop computer configuration. The average elapsed time (elapsed_mean) and standard deviation (elapsed_sd) of ten runs is reported in the table. The single chunk was only able to run for the smallest dataset size (N = 75,000). We used $k = 15$ and used a batch size of $b = 500$ observations.

chunk_size	dim_1	dim_2	ncells	ngenes	batch	memory	elapsed_mean	elapsed_sd
per cell (best)	5000	1	75000	5000	500	0.77	1.46	0.03
per cell (best)	5000	1	150000	5000	500	0.90	1.43	0.02
per cell (best)	5000	1	300000	5000	500	0.96	2.91	0.04
per cell (best)	5000	1	500000	5000	500	1.23	4.94	0.02
per cell (best)	5000	1	750000	5000	500	1.33	8.27	0.29
per cell (best)	5000	1	1000000	5000	500	1.52	9.84	0.33
default	258	3872	75000	5000	500	0.97	1.82	0.19
default	182	5477	150000	5000	500	1.04	2.71	0.44
default	129	7745	300000	5000	500	1.12	5.90	1.18
default	100	10000	500000	5000	500	1.28	9.87	1.68
default	81	12247	750000	5000	500	1.45	15.73	2.68
default	70	14142	1000000	5000	500	1.84	19.86	2.95
per gene (worst)	1	75000	75000	5000	500	1.03	1.36	0.11
per gene (worst)	1	150000	150000	5000	500	1.15	2.74	0.36
per gene (worst)	1	300000	300000	5000	500	1.25	5.41	0.49
per gene (worst)	1	500000	500000	5000	500	1.58	10.15	1.31
per gene (worst)	1	750000	750000	5000	500	2.49	16.50	1.31
per gene (worst)	1	1000000	1000000	5000	500	3.73	25.20	2.93
single chunk	5000	75000	75000	5000	500	6.39	1.97	NA
