# Supplementary material for: mbkmeans: Fast clustering for single cell data using mini-batch k-means
Source: PLoS Comput Biol. 2021 Jan 26;17(1):e1008625. doi: 10.1371/journal.pcbi.1008625 (PMC7864438; doi:10.1371/journal.pcbi.1008625)
Supplement: S5 Table — (PDF) [file pcbi.1008625.s021.pdf]

S5 Table Computational time for each of the steps of the pipeline for the full 1.3 million mouse brain cells

Step	Package	Compute Time
Preprocessing	*scater*	8 mins
Clustering, All Genes	*mbkmeans*	8.5 mins
Normalization	*scran*	5.18 hrs
PCA	*BioSingular*	96 hrs
Clustering, Reduced Dims (16 times to determine k)	*mbkmeans*	3 mins
Visualization	UMAP	20 mins
	TSNE	2.5 hrs
Total		104 hrs
