# Supplementary material for: mbkmeans: Fast clustering for single cell data using mini-batch k-means
Source: PLoS Comput Biol. 2021 Jan 26;17(1):e1008625. doi: 10.1371/journal.pcbi.1008625 (PMC7864438; doi:10.1371/journal.pcbi.1008625)
Supplement: S6 Table — (PDF) [file pcbi.1008625.s022.pdf]

S6 Table Identification of *mbkmeans* clusters with Marker Genes

mbkmeans Cluster	Genes	Biological Subtype	References
1	*Mdk, Mki67, Nde1*	Radial precursor / Proliferating	[1,2]
2	*Gad1, Gad2, Foxp2, Syt6, Bcl11b*	Interneurons	[2–4]
3	*Neurod1, Synpr, Prox1*	Granule Cells	[5]
4	*Sox4, Sox11, Meis2*	Pyramidal progenitors	[5]
5	*Gad1, Gad2, Sst, Lhx6*	Interneurons	[2–4]
6	*Meis2, Neurod6*	Pyramidal progenitors	[5]
7	*Vim, Fabp7, Sox9, Dbi*	Radial precursor	[1]
8	*Crym, Tnr, Cck*	Pyramidal neurons	[2,3]
9	*Sstr2, Mdk, Eomes*	Neuronal progenitors	[1,2]
10	*Pdgfra, Vim, Dbi*	OPC	[5]
11	*Cck, Ntf3, Nr4a2*	Pyramidal neurons	[3–5]
12	*Rgs5, Igfbp7*	Endothelial/SMC/Mural	[2,5,6]
13	*Neurod6, Satb2, Meis2*	Pyramidal progenitors	[5]
14	*Hbb−bs, Hba−a1, Hba−a2*	Blood	
15	*Reln, Pcp4, Tbr1*	Cajal-Retzius cells	[7]

References

[1] Yuzwa SA, Borrett MJ, Innes BT, Voronova A, Ketela T, Kaplan DR, et al. Developmental emergence of adult neural stem cells as revealed by single-cell transcriptional profiling. Cell reports. 2017;21(13):3970–3986.

[2] Loo L, Simon JM, Xing L, McCoy ES, Niehaus JK, Guo J, et al. Single-cell transcriptomic analysis of mouse neocortical development. Nature communications. 2019;10(1):1–11.

[3] Tasic B, Yao Z, Graybuck LT, Smith KA, Nguyen TN, Bertagnolli D, et al. Shared and distinct transcriptomic cell types across neocortical areas. Nature. 2018;563(7729):72–78.

[4] Zeisel A, Muñoz-Manchado AB, Codeluppi S, Lönnerberg P, La Manno G, Juréus A, et al. Cell types in the mouse cortex and hippocampus revealed by single-cell RNA-seq. Science. 2015;347(6226):1138–1142.

[5] Rosenberg AB, Roco CM, Muscat RA, Kuchina A, Sample P, Yao Z, et al. Single-cell profiling of the developing mouse brain and spinal cord with split-pool barcoding. Science. 2018;360(6385):176–182.

[6] Saunders A, Macosko EZ, Wysoker A, Goldman M, Krienen FM, de Rivera H, et al. Molecular diversity and specializations among the cells of the adult mouse brain. Cell. 2018;174(4):1015–1030.

[7] Bhaduri A, Nowakowski TJ, Pollen AA, Kriegstein AR. Identification of cell types in a mouse brain single-cell atlas using low sampling coverage. BMC biology. 2018;16(1):1–10.
