# Supplementary material for: mbkmeans: Fast clustering for single cell data using mini-batch k-means
Source: PLoS Comput Biol. 2021 Jan 26;17(1):e1008625. doi: 10.1371/journal.pcbi.1008625 (PMC7864438; doi:10.1371/journal.pcbi.1008625)
Supplement: S7 Table — SNN: Shared Nearest Neighbors; BBKNN: Batch-Balanced K-Nearest Neighbors. (PDF) [file pcbi.1008625.s023.pdf]

S7 Table Computational time for graph-based clustering. SNN: Shared Nearest Neighbors; BBKNN: Batch-Balanced K-Nearest Neighbors.

Method	Step	Package	Compute Time
**SNN + Louvain**			
(50 PCs, Annoy Approx)			
	SNN Graph	*BiocNeighbors*	7.5 mins
	Louvain Clustering	*BiocNeighbors*	28 mins
Total			**35.5 mins**
**SNN + Louvain**			
(50 PCs, Exact)			
	SNN Graph	*BiocNeighbors*	128 mins
	Louvain Clustering	*BiocNeighbors*	28 mins
Total			**156 mins**
**BBKNN + Leiden**			
(50 PCs, Annoy Approx)			
	BBKNN	*scanpy*	1.5 mins
	Leiden Clustering	*scanpy*	47 mins
Total			**48.5 mins**
**SNN + Louvain**			
(All Genes, Annoy Approx)			
	SNN Graph	*BiocNeighbors*	9.9 hrs
	Louvain Clustering	*BiocNeighbors*	28 mins
Total			**10.4 hrs**
