# Supplementary material for: mbkmeans: Fast clustering for single cell data using mini-batch k-means
Source: PLoS Comput Biol. 2021 Jan 26;17(1):e1008625. doi: 10.1371/journal.pcbi.1008625 (PMC7864438; doi:10.1371/journal.pcbi.1008625)
Supplement: S1 File — (GZ) [file pcbi.1008625.s024.tar.gz › mbkmeans/inst/doc/Vignette.html]

An introduction to mbkmeans


# An introduction to mbkmeans

Yuwei Ni, Davide Risso, Stephanie Hicks, and Elizabeth Purdom

#### Last modified: November 7, 2020; Compiled: November 13, 2020

# Contents

- 1 Installation
- 2 Introduction
  - 2.1 Example dataset
- 3 `mbkmeans`
  - 3.1 Choice of arguments
    - 3.1.1 Batch size
    - 3.1.2 Initialization
  - 3.2 Running `mbkmeans` with multiple values of \(k\)
- 4 Comparison with *k*-means
- 5 Use with bluster
- 6 Working with on-disk data in Bioconductor
- References

# 1 Installation

To install the package, please use the following.

```
if (!requireNamespace("BiocManager"))
    install.packages("BiocManager")
BiocManager::install("mbkmeans")
```

# 2 Introduction

This vignette provides an introductory example on how
to work with the `mbkmeans` package, which contains an
implementation of the mini-batch k-means algorithm
proposed in (Sculley 2010) for large single-cell
sequencing data. This algorithm runs the \_k\_means iterations on small, random subsamples of data (``mini batches’’) rather than the entire dataset. This both speeds up computation and reduces the amount of data that must be contained in memory.

The main function to be used by the users is `mbkmeans`.
This is implemented as an S4 generic and methods are
implemented for `matrix`, `Matrix`, `HDF5Matrix`,
`DelayedMatrix`, `SummarizedExperiment`, and
`SingleCellExperiment`.

Most of this work was inspired by the
`MiniBatchKmeans()` function implemented in the
`ClusterR` R package and we re-use many of the
C++ functions implemented there.

Our main contribution here is to provide an
interface to the `DelayedArray` and `HDF5Array`
packages so that `mbkmeans` algorithm only brings into memory at any time the “mini-batches” needed for the current iteration, and never requires loading the entire dataset into memory. This allows the user to run the mini-batch
*k*-means algorithm on very large datasets that do not fit
entirely in memory. `mbkmeans` also runs seamlessly on in-memory matrices provided by the user, and doing so significantly increases the speed of the algorithm compared to the standard *k*-means algorithm ((Hicks et al. 2020)). A complete comparison of `mbkmeans` with other implementations can be found in (Hicks et al. 2020).

The motivation for this work is the clustering of
large single-cell RNA-sequencing (scRNA-seq) datasets,
and hence the main focus is on Bioconductor’s
`SingleCellExperiment`and `SummarizedExperiment`
data container. For this reason, `mbkmeans` assumes a
data representation typical of genomic data, in which
genes (variables) are in the rows and cells
(observations) are in the column. This is contrary to
most other statistical applications, and notably to
the `stats::kmeans()` and `ClusterR::MiniBatchKmeans()`
functions that assume observations in rows.

We provide a lower-level `mini_batch()` function that
expects observations in rows and is expected to be a direct
replacement of `ClusterR::MiniBatchKmeans()` for on-disk
data representations such as `HDF5` files. The rest of
this document shows the typical use case through the
`mbkmeans()` interface; users interested in the
`mini_batch()` function should refer to its manual page.

## 2.1 Example dataset

To illustrate a typical use case, we use the
`pbmc4k` dataset of the
`TENxPBMCData` package.
This dataset contains a set of about 4,000 cells from
peripheral blood from a healthy donor and is expected
to contain many types or clusters of cell.

We would note that `mbkmeans` is designed for very large datasets, in particular for datasets large enough that either the full data matrix can’t be held in memory, or the computations necessary cannot be computed. This vignette works with a small dataset to allow the user to quickly follow along and understand the commands. (Hicks et al. 2020) shows the example of using `mbkmeans` with a dataset with 1.3 million cells, which is a more appropriate size for observing improved memory usage and improved speed.

First, we load the needed packages.

```
library(TENxPBMCData)
library(scater)
library(SingleCellExperiment)
library(mbkmeans)
library(DelayedMatrixStats)
```

Note that in this vignette, we do not aim at identifying
biologically meaningful clusters (that would
entail a more sophisticated normalization of data and
dimensionality reduction), but instead we only aim to show
how to run mini-batch *k*-means on a HDF5-backed
matrix.

We normalize the data simply by scaling for the total
number of counts using `scater` and select the 1,000
most variable genes and a random set of 100 cells to
speed-up computations.

```
tenx_pbmc4k <- TENxPBMCData(dataset = "pbmc4k")

set.seed(1034)
idx <- sample(seq_len(NCOL(tenx_pbmc4k)), 100)
sce <- tenx_pbmc4k[, idx]

#normalization
sce <- logNormCounts(sce)

vars <- rowVars(logcounts(sce))
names(vars) <- rownames(sce)
vars <- sort(vars, decreasing = TRUE)

sce1000 <- sce[names(vars)[1:1000],]

sce1000
```

```
## class: SingleCellExperiment 
## dim: 1000 100 
## metadata(0):
## assays(2): counts logcounts
## rownames(1000): ENSG00000090382 ENSG00000204287 ... ENSG00000117748
##   ENSG00000135968
## rowData names(3): ENSEMBL_ID Symbol_TENx Symbol
## colnames: NULL
## colData names(12): Sample Barcode ... Date_published sizeFactor
## reducedDimNames(0):
## altExpNames(0):
```

# 3 `mbkmeans`

The main function, `mbkmeans()`, returns a
list object including `centroids`,
`WCSS_per_cluster` (where WCSS stands for
within-cluster-sum-of-squares),
`best_initialization`, `iters_per_initiazation`
and `Clusters`.

It takes any matrix-like object as input, such
as `SummarizedExperiment`, `SingleCellExperiment`,
`matrix`, `DelayedMatrix` and `HDF5Matrix`.

In this example, the input is a `SingleCellExperiment`
object.

```
res <- mbkmeans(sce1000, clusters = 5,
                reduceMethod = NA,
                whichAssay = "logcounts")
```

The number of clusters (such as *k* in the
*k*-means algorithm) is set through the `clusters`
argument. In this case, we set `clusters = 5` for
no particular reason. For `SingleCellExperiment`
objects, the function provides the `reduceMethod`
and `whichAssay` arguments. The `reduceMethod` argument
should specify the dimensionality reduction slot
to use for the clustering, and the default is
“PCA”. Note that this *does not perform* PCA but
only looks at a slot called “PCA” already stored
in the object. Alternatively, one can specify
`whichAssay` as the assay to use as input to
mini-batch *k*-means. This is used only when
`reduceMethod` option is `NA`. See `?mbkmeans`
for more details.

## 3.1 Choice of arguments

There are additional arguements in `mbkmeans()`
that make the function more flexible
and suitable for more situations.

### 3.1.1 Batch size

The size of the mini batches is set through the
`batch_size` argument. This argument gives the size of the samples of data brought into memory at any one time, and thus constrains the memory usage of the alogrithm.

The `blocksize()` function can be used to set the batch size to the maximum allowed by the available memory.
It considers both the number of columns in the dataset
and the amount of RAM on the current matchine to
calculate as large of a batch size as reasonable for
the RAM available to the session. The calculation uses
`get_ram()` function in `benchmarkme` package. See
the `benchmarkme` vignette for more details.

```
batchsize <- blocksize(sce1000)
batchsize
```

```
## [1] 100
```

In this simple example, because the whole data fits in memory,
the default batch size would be a single
batch of size 100.

(Hicks et al. 2020) provides a comparison of the effect of batch size for large datasets (i.e. where the full data cannot be stored in memory). Their results show the algorithm to be robust to a large range of batch sizes (1,000-10,000 cells): accuracy of the results were equivalent if the batch size was above 500-1000 cells, with no noticable advantage in memory usage or speed as long as the batches were smaller than roughly 10,000 cells (corresponding to a subsample of the full data matrix whose memory foot print is not noticeable on most modern computers). For this reason, we would err on the larger range of batch size suggested in (Hicks et al. 2020) – 10,000 cells. Furthermore, since the initialization uses by default the same number of cells as batch size, this gives a robust sample for determining the initial start point.

We would note that it is better to make choices on batch size based on choosing the *absolute* number of cells in a batch rather than choosing batch size based on a percentage of the overall sample size. If batch size is chosen based on the percentage of cells, very large datasets will use very large batches of cells, negating the memory and speed gains. Moreover such large batches are not needed for good performance of the algorithm. The continual resampling of the batches works like stochastic gradient descent and allows for (local) minimization of the objective function (Sculley 2010) with relatively small batch sizes, as demonstrated on single-cell data in the work of (Hicks et al. 2020). If anything, the size of the batch should be governed more by the complexity of the problem (e.g. the number of underlying true clusters or subtypes), rather than the number of overall cells in the dataset.

### 3.1.2 Initialization

The performance of mini-batch *k*-means greatly
depends on the process of initialization. We
implemented two different initialization methods:

1. Random initialization, as in regular *k*-means;
2. `kmeans++`, as proposed in (Arthur and Vassilvitskii 2007). The default is “kmeans++”.

The percentage of data to use for the initialization
centroids is set through the `init_fraction` argument,
which should be larger than 0 and less than 1. The
default value is given so that the proportion matches the value of `batch_size`, converted to a proportion of the data.

```
res_random <- mbkmeans(sce1000, clusters = 5, 
                reduceMethod = NA,
                whichAssay = "logcounts",
                initializer = "random")
table(res$Clusters, res_random$Clusters)
```

```
##    
##      1  2  3  4  5
##   1  0  0  0  0  1
##   2  7 12  0  2  0
##   3  9  0 41  0  0
##   4  0  0  0  0 27
##   5  0  0  1  0  0
```

Note that large values of `init_fraction` will result in that proportion of the data being held in memory and used in the initialization of the `kmeans++` algorithm. Therefore, large values of `init_fraction` will require a great deal of memory – potentially more than that used by the actual clustering algorithm. Indeed keeping the fraction used constant for larger and larger datasets will result in commiserate numbers of cells being used in the initialization and increasing the memory usage (similar to the issues discussed above for batch size). Therefore, we recommend that users keep the default and make changes to the `batch_size` parameter as desired, ensuring consistent memory usage across both the initialization stage and the actual clustering stage of the algorithm. As mentioned in the discussion above on `batch_size`, for this reason we would recommend `batch_size` on the larger range suggested as reasonable in (Hicks et al. 2020) so as to improve the initialization.

## 3.2 Running `mbkmeans` with multiple values of \(k\)

The main parameter to set in \(k\)-means and its variants is the number of clusters \(k\). `mbkmeans` is quick enough to rerun the clustering algorithm with different number of clusters even in very large datasets.

Here, we apply `mbkmeans` with \(k\) from 5 to 15 and select the number of clusters using the elbow method (i.e., the value that corresponds to the point of inflection on the curve). We note that this is just a rule of thumb for selecting the number of clusters, and many different methods exist to decide the appropriate value of \(k\).

To speed up the computations, we will cluster on the top 20 PCs.

```
sce1000 <- runPCA(sce1000, ncomponents=20)

ks <- seq(5, 15)
res <- lapply(ks, function(k) {
    mbkmeans(sce1000, clusters = k,
             reduceMethod = "PCA",
             calc_wcss = TRUE, num_init=10)
})

wcss <- sapply(res, function(x) sum(x$WCSS_per_cluster))
plot(ks, wcss, type = "b")
```

From the plot, it seems that `k = 12` could be a reasonable value.

# 4 Comparison with *k*-means

Note that if we set `init_fraction = 1`,
`initializer = "random"`, and `batch_size = ncol(x)`,
we recover the classic *k*-means algorithm.

```
res_full <- mbkmeans(sce1000, clusters = 5,
                     reduceMethod = NA,
                     whichAssay = "logcounts",
                     initializer = "random",
                     batch_size = ncol(sce1000))
res_classic <- kmeans(t(logcounts(sce1000)), 
                      centers = 5)
table(res_full$Clusters, res_classic$cluster)
```

```
##    
##      1  2  3  4  5
##   1 28  0  0  0  0
##   2  0  0  0  0 14
##   3  0  7  6  0  0
##   4  0  0  0  1  0
##   5  0  0  9 35  0
```

Note however, that since the two algorithms start from different random initializations, they will not always converge to the same solution. Furthremore, if the algorithm uses batches smaller than the full dataset (`batch_size<ncol(x)`), then `mbkmeans` will not converge to the same result as `kmeans` even with the same initialization. This is due to the fact that `kmeans` and `mbkmeans` have different search paths for an optimum and the problem is non-convex.

For a comparison of memory usage, speed, and accuracy of `mbkmeans` with `kmeans` for the non-trivial case where the algorithm uses batches, see (Hicks et al. 2020), where it is demonstrated that `mbkmeans` provides significant speed improvements and uses less memory than `kmeans`, without loss of accuracy.

# 5 Use with bluster

The *bluster* package provides a flexible and extensible framework for clustering in Bioconductor packages/workflows.
The `clusterRows()` function controls dispatch to different clustering algorithms.
In this case, we will use the mini-batch *k*-means algorithm to cluster cells into cell populations based on their principal component analysis (PCA) coordinates.

```
mat <- reducedDim(sce1000, "PCA")
dim(mat)
```

```
## [1] 100  20
```

In the following three scenarios, we pass the number of cluster centers as a number using the `centers=5` argument.
In addition, we can also pass other `mbkmeans` arguments (e.g. `batch_size=10`).
In the first two cases, we only return the cluster labels.
However, in the third scenario, we ask for the full `mbkmeans` output.

```
clusterRows(mat, MbkmeansParam(centers=5))
```

```
##   [1] 1 1 5 3 5 5 3 4 4 3 5 2 1 4 4 4 3 5 4 3 1 4 5 5 4 3 4 4 5 4 3 5 1 4 2 4 1
##  [38] 5 5 5 5 5 5 5 5 3 4 3 1 5 5 2 4 3 2 5 1 4 1 3 5 5 4 4 3 5 1 1 5 5 4 5 5 4
##  [75] 5 3 4 5 3 4 1 4 5 4 2 5 1 5 4 4 5 4 1 1 5 5 2 5 4 2
## Levels: 1 2 3 4 5
```

```
clusterRows(mat, MbkmeansParam(centers=5, batch_size=10))
```

```
##   [1] 3 3 3 2 3 3 2 4 5 2 3 1 3 4 4 4 2 3 4 2 3 4 3 3 4 2 4 5 3 4 2 3 3 4 1 4 3
##  [38] 3 3 3 3 3 3 3 3 2 5 2 3 3 3 1 5 2 1 3 3 5 3 2 3 3 4 5 2 3 3 3 3 3 4 3 3 4
##  [75] 3 2 5 3 2 4 3 5 3 4 1 3 3 3 4 5 3 4 3 3 3 3 3 3 5 1
## Levels: 1 2 3 4 5
```

```
clusterRows(mat, MbkmeansParam(centers=5, batch_size=10), full = TRUE)
```

```
## $clusters
##   [1] 4 4 2 3 2 2 3 5 5 3 2 1 4 5 5 5 3 2 5 3 4 5 2 2 5 3 5 5 2 5 3 2 2 5 1 5 4
##  [38] 2 2 2 2 2 2 2 2 3 5 3 4 2 2 1 5 3 1 2 2 5 2 3 2 2 5 5 3 2 4 2 2 2 5 2 2 5
##  [75] 2 3 5 2 3 5 4 5 2 5 1 2 4 2 5 5 2 5 4 4 2 2 4 2 5 4
## Levels: 1 2 3 4 5
## 
## $objects
## $objects$centroids
##            [,1]       [,2]       [,3]      [,4]       [,5]       [,6]
## [1,] -0.4577116 17.6859771  7.2125414  6.146455 -0.1617895  3.6025364
## [2,] -7.3532260 -2.2780222 -4.5269148  1.899054  2.3079454  1.3317261
## [3,] -0.6271520 -6.4798245  9.0902508 -1.570689  0.8962586 -0.3283511
## [4,] -5.3909583  7.9839453  1.0851376 -2.136876 -2.2299752 -3.8418710
## [5,] 13.4092572  0.2561473 -0.8165787 -1.668530  0.2572103  0.2130437
##            [,7]       [,8]       [,9]      [,10]      [,11]      [,12]
## [1,] -0.4362298 -1.4929562 -0.6043261  2.8130528  0.7867966  0.1072129
## [2,] -1.7401923  0.3085988 -0.4275444 -1.9503845 -0.9773493  2.1148466
## [3,] -1.7672493  0.6408476  2.5692173  0.4198125 -0.6886444 -1.0662452
## [4,]  0.1413952  3.6700235 -3.1699755  1.6901565 -2.2607733  0.7971820
## [5,]  0.2421881  0.1048978 -0.2709161 -0.3203373  0.2076287 -1.0766293
##           [,13]       [,14]       [,15]       [,16]      [,17]       [,18]
## [1,]  0.2503804  3.22734394 -0.61769167 -2.49754022 -1.2880616  2.30317564
## [2,] -0.2037572 -0.19111259 -0.11096431 -0.76458340  0.8955021  0.26083966
## [3,] -1.3175808  1.03245658  0.20983011  0.04326286 -0.4047900  0.71365303
## [4,] -1.0580787 -0.12535594  1.45397730  0.33997729  1.7561255 -1.00222783
## [5,]  0.9634335 -0.03337336  0.04601148  1.33055069 -1.3651570  0.02862997
##           [,19]       [,20]
## [1,] -1.0135887 -1.61511808
## [2,] -1.0779235  0.73646774
## [3,] -0.3561008 -0.40158727
## [4,]  1.2728415 -2.50454614
## [5,]  0.7295523 -0.05236506
## 
## $objects$WCSS_per_cluster
## numeric(0)
## 
## $objects$best_initialization
## [1] 1
## 
## $objects$iters_per_initialization
##      [,1]
## [1,]   23
## 
## $objects$Clusters
##   [1] 4 4 2 3 2 2 3 5 5 3 2 1 4 5 5 5 3 2 5 3 4 5 2 2 5 3 5 5 2 5 3 2 2 5 1 5 4
##  [38] 2 2 2 2 2 2 2 2 3 5 3 4 2 2 1 5 3 1 2 2 5 2 3 2 2 5 5 3 2 4 2 2 2 5 2 2 5
##  [75] 2 3 5 2 3 5 4 5 2 5 1 2 4 2 5 5 2 5 4 4 2 2 4 2 5 4
```

While many of the times clustering will be performed on in-memory data (for example after PCA), the `clusterRows` function also accepts any matrix-like object (in-memory or on-disk data representation) that `mbkmeans` accepts.

For example, we can cluster using `mbkmeans` on the `logcounts` assay in the `sce1000` object (similar to above), which is a `DelayedMatrix` object from the *DelayedArray* package.

**Note**: We transpose the matrix as the `clusterRows` function expects observations along the rows and variables along the columns.

```
logcounts(sce1000)
```

```
## <1000 x 100> matrix of class DelayedMatrix and type "double":
##                      [,1]      [,2]      [,3] ...     [,99]    [,100]
## ENSG00000090382  0.000000  1.482992  0.000000   . 5.4330604 0.0000000
## ENSG00000204287  2.198815  0.000000  0.000000   . 5.3387971 0.0000000
## ENSG00000163220  1.762993  1.482992  0.000000   . 2.1176311 2.0642508
## ENSG00000143546  0.000000  1.135438  1.113099   . 0.3825221 0.0000000
## ENSG00000019582  3.402730  1.762861  1.733874   . 6.2009459 2.0642508
##             ...         .         .         .   .         .         .
## ENSG00000145779 0.0000000 0.6766523 0.0000000   . 0.9342195 0.0000000
## ENSG00000106948 0.0000000 1.1354382 0.0000000   . 0.9342195 1.3735556
## ENSG00000155660 0.0000000 0.6766523 0.0000000   . 0.3825221 1.3735556
## ENSG00000117748 0.0000000 0.0000000 0.0000000   . 0.0000000 0.0000000
## ENSG00000135968 0.0000000 0.0000000 0.0000000   . 0.3825221 0.0000000
```

```
clusterRows(t(logcounts(sce1000)), MbkmeansParam(centers=4))
```

```
##   [1] 4 4 4 3 4 4 3 2 2 3 4 4 4 2 2 2 3 4 2 3 4 2 4 4 2 3 2 2 4 2 3 4 4 2 4 2 4
##  [38] 4 4 4 4 4 4 4 4 3 2 3 4 4 4 4 2 3 4 4 4 2 4 3 4 4 2 2 3 4 4 4 1 4 2 4 4 2
##  [75] 4 3 2 4 3 2 4 2 4 2 4 4 4 4 2 2 4 2 4 4 4 4 4 4 2 4
## Levels: 1 2 3 4
```

# 6 Working with on-disk data in Bioconductor

This vignette is focused on the use of the `mbkmeans` package.
For the users interested in learning more about working with `HDF5` files and on-disk data in Bioconductor, we recommend the excellent DelayedArray workshop, presented by Peter Hickey at Bioc2020.

# References

Arthur, David, and Sergei Vassilvitskii. 2007. “K-Means++: The Advantages of Careful Seeding.” In *Proceedings of the Eighteenth Annual Acm-Siam Symposium on Discrete Algorithms*, 1027–35. Society for Industrial; Applied Mathematics.

Hicks, Stephanie C., Ruoxi Liu, Yuwei Ni, Elizabeth Purdom, and Davide Risso. 2020. “Mbkmeans: Fast Clustering for Single Cell Data Using Mini-Batch K-Means.” *bioRxiv*. https://doi.org/10.1101/2020.05.27.119438.

Sculley, David. 2010. “Web-Scale K-Means Clustering.” In *Proceedings of the 19th International Conference on World Wide Web*, 1177–8. ACM.
