# Supplementary material for: mbkmeans: Fast clustering for single cell data using mini-batch k-means
Source: PLoS Comput Biol. 2021 Jan 26;17(1):e1008625. doi: 10.1371/journal.pcbi.1008625 (PMC7864438; doi:10.1371/journal.pcbi.1008625)
Supplement: S1 Text — (PDF) [file pcbi.1008625.s025.pdf]

Supplementary text for mbkmeans: fast clustering for single cell data using mini-batch k-means

Stephanie C. Hicks[1], Ruoxi Liu[2], Yuwei Ni[3#], Elizabeth Purdom[4], Davide Risso[5*]

1 Department of Biostatistics, Johns Hopkins Bloomberg School of Public Health, Baltimore, Maryland, USA
2 Department of Applied Mathematics and Statistics, Johns Hopkins University, Baltimore, Maryland, USA
3 Department of Healthcare Policy and Research, Weill Cornell Medical College, New York, New York, USA
4 Department of Statistics, University of California, Berkeley, Berkeley, California, USA
5 Department of Statistical Sciences, University of Padova, Padova, Italy

# Current Address: R4 Technologies Inc., 38 Grove Street, Ridgefield, CT, 06877, USA
* risso.davide@gmail.com

Simulated gene expression data

We simulated gene expression data $Y_{i,g}$ with $i \in (1, \ldots, N)$ cells (or observations) and $g \in (1, \ldots G)$ genes (or features) representing normalized gene expression data in the following way:

(i) Assume there are k true clusters with probabilities $p_1, \ldots, p_k$. We randomly sample (without replacement) the cluster label for i^{th} observation $Z_i \in (1, \ldots, k)$ with probabilities $p_1, \ldots, p_k$.

(ii) For the ith observation with the kth cluster label, we assume the true biological structure is sampled from a bivariate normal distribution $X_{ik} \sim N_2(\boldsymbol{\mu}_k, \boldsymbol{\Sigma}_k)$ where $\boldsymbol{\mu}_k = (\mu_{1k}, \mu_{2k})$, $\text{diag}(\boldsymbol{\Sigma}_k) = (\sigma^2_{1k}, \sigma^2_{2k})$ and zero in off diagonals. We combine the N observations into a $(N, 2)$-dimensional matrix $\mathbf{X}_{(N,2)}$, which is made up of a mixture of k bivariate normal distributions.

(iii) Next, we simulate data from a normal distribution $\mathbf{Z}_{(G,2)} \sim N(0, 1)$, to project the N observations currently in a 2-dimensional space into a G-dimensional space to mimic high-dimensional gene expression data, or $\mathbf{A}_{(N,G)} = \mathbf{X}\mathbf{Z}^T$.

(iv) Finally, we add random noise $\boldsymbol{\varepsilon}_{(N,G)} \sim N(0, 1)$ to obtain the simulated normalized gene expression data $\mathbf{Y}_{(N,G)} = (\mathbf{Y}_1, \ldots, \mathbf{Y}_N)$, or $\mathbf{Y} = \mathbf{A} + \boldsymbol{\varepsilon}$.

A scRNA-seq experiment with 1.3 million mouse brain cells

A scRNA-seq experiment was performed with the 10X Chromium Genomics platform [1] measuring the gene expression in mouse cells that came from three regions of the brain (cortex, hippocampus, and subventricular zone) and two mouse embryos (E18 C57BL/6

mice). This resulted in a dataset containing $G = 27{,}998$ genes and $N = 1{,}306{,}127$ cells. The data can be downloaded as a sparse HDF5 file directly from the 10X website (https://support.10xgenomics.com/single-cell-gene-expression/datasets/1.3.0/1M_neurons).

However, we used the *TENxBrainData* Bioconductor data package [2], which stores the data as a dense matrix in a HDF5 file. The package returns an object of the `SingleCellExperiment` class [3]. We calculated quality control metrics using the `calculateQCMetrics` function from the *scater* Bioconductor package [4]. We then applied a cell-filter to remove cells with a high proportion of mitochondrial reads (at least 3 median absolute deviations away from the median). We also applied a gene-filter to keep only the genes with at least 1 UMI in at least 1% of the cells. This filtering procedure resulted in a final matrix of 11,720 genes and 1,232,055 cells. Finally, we subsampled cells (random sampling without replacement) and saved each downsampled and pre-processed data as a new HDF5-based `SingleCellExperiment` object. We used all 11,720 genes for the full analysis, while we focused on the 5,000 most variable genes for the subsampling analysis. Since we need fast access to the columns (cells) of the matrix, we saved the data with chunks of dimension $(1 \times G)$, (i.e., each chunk contained all gene expression measurements for one cell) with default compression level. Unless noted otherwise, we used this chunk geometry to perform all the analyses. In Figure 4, we explore the impact of the HDF5 chunk geometry on the performance of *mbkmeans*.

In the full analysis, we clustered both the $11{,}720 \times 1{,}232{,}055$ matrix of gene expression and the $1{,}232{,}055 \times 50$ matrix of the top 50 principal components with *mbkmeans* and Louvain clustering. For Louvain clustering, we used the `buildSNNGraph` function (with default number of neighbors `k=10`, using 6 cores) of the *scran* package to create a Shared Nearest Neighbor graph, using the Annoy algorithm for approximate nearest neighbor identification (`BNPARAM=AnnoyParam()`) and the exact nearest-neighbor search with the KMKNN algorithm (`BNPARAM=KmknnParam()`). We then used the `cluster_louvain` function of the *igraph* package to perform the clustering.

To compare our approach to a *scanpy* workflow, we adapted a tutorial provided by the authors of *scanpy* at `https://github.com/theislab/scanpy_usage/tree/master/170522_visualizing_one_million_cells`. We used the `scanpy.external.pp.bbknn` function (with default parameters) instead of `scanpy.pp.neighbors` to allow batch correction and the `scanpy.tl.leiden` (with default parameters) function instead of `scanpy.tl.louvain` to use Leiden clustering. A Jupyter Notebook with our analysis can be found at `https://github.com/stephaniehicks/benchmark-hdf5-clustering/tree/master/main/python`.

References

1. Zheng GXY, Terry JM, Belgrader P, Ryvkin P, Bent ZW, Wilson R, et al. Massively parallel digital transcriptional profiling of single cells. Nat Commun. 2017;8:14049. doi:10.1038/ncomms14049.

2. Lun A, Morgan M. TENxBrainData: Data from the 10X 1.3 Million Brain Cell Study; 2019.

3. Amezquita RA, Lun ATL, Becht E, Carey VJ, Carpp LN, Geistlinger L, et al. Orchestrating single-cell analysis with Bioconductor. Nat Methods. 2019;doi:10.1038/s41592-019-0654-x.

4. McCarthy DJ, Campbell KR, Lun ATL, Wills QF. Scater: pre-processing, quality control, normalization and visualization of single-cell RNA-seq data in R. Bioinformatics. 2017;33(8):1179–1186. doi:10.1093/bioinformatics/btw777.
