# Supplementary material for: mbkmeans: Fast clustering for single cell data using mini-batch k-means
Source: PLoS Comput Biol. 2021 Jan 26;17(1):e1008625. doi: 10.1371/journal.pcbi.1008625 (PMC7864438; doi:10.1371/journal.pcbi.1008625)
Supplement: Attachment — Submitted filename: mbkmeans - response to reviewers.pdf [file pcbi.1008625.s026.pdf]

General comments

We would like to thank the editor and the three reviewers for their careful evaluation of our work.

We provide below a point-by-point response to their critiques, but first we summarize the main changes made with respect to the first version of the manuscript.

In the revised version of our work, we now (1) highlight the novelty of our work with respect to existing approaches and (2) compare our method to state-of-the-art algorithms for the clustering of single-cell RNA-seq.

As for (1), we have included comparisons with an existing implementation of the mini-batch k-means clustering, implemented in the *ClusterR* R package. We show that even our in-memory implementation is more memory efficient without loss of accuracy and speed (new Figure S1, S2, and S7). More importantly, *mbkmeans* is the first implementation of mini-batch k-means that takes full advantage of out-of-memory data and of the HDF5 file-format, allowing us to analyze extremely large data matrices. For a comparison, while *ClusterR*'s implementation uses more than 40 GB of RAM for 750k cells, our in-memory implementation uses less than 30GB and our out-of-memory implementation uses 1.4 GB, allowing us to successfully analyze 1M cells on a 64GB RAM machine, unlike *ClusterR* (Figure S1).

As for (2), we have included comparisons with two of the most popular and most computationally efficient approaches currently in use for the clustering of single-cell RNA-seq, namely Louvain and Leiden clustering, as implemented in R and Python, respectively. Both methods are substantially slower and less memory efficient than *mbkmeans*, taking almost 3 hours to cluster the full 1.3 Million cells data matrix (compared to 8.5 minutes for *mbkmeans*) and 35-48 minutes to cluster the 1.3 Million cells in the space of the top 50 principal components (compared to 30 seconds for *mbkmeans*). These results are summarized in the new Table S7. Furthermore, the resulting partitions are qualitatively similar (see new Figures S15 and S16).

Finally, a new, updated version of the package vignette is now available as part of the release version (currently 1.6.1) of the *mbkmeans* package. This new, extended vignette provides more context for the package, guidelines for the choice of the parameters, and examples on how to run specific clustering tasks (e.g., running the method with multiple values of *k* and choosing the most appropriate one).

We believe that, taken together, these changes demonstrate the novelty and competitiveness of *mbkmeans* for the clustering of large single-cell datasets and address all the comments raised by the reviewers.

Reviewer comments

Reviewer #1:

Hicks et al present "mbkmeans: fast clustering for single cell data using mini-batch k-means". The paper is very well written and looks very logical. mbkmeans development is very timely, since the size of scRNAseq data has grown dramatically in the last few years. mbkmeans solves the problem of in memory clustering by operating with data on disk due to utilisation of hdf5 format. The package is already available on bioconductor and is well integrated with the bioconductor universe. The datasets used are of appropriate size and quality. I recommend this paper for publication.

We thank the reviewer for their positive comments and their enthusiasm for our paper.

Reviewer #2:

mbkmeans (multi-batch K-means) is a package that fills a gap in the R/Bioconductor ecosystem: tractable data integration of total mRNA copy number estimates from millions of cells, by k-means clustering. As such, alternatives that exist for comparison appear to require stepping out of the controlled, vetted ecosystem into something like scanpy or bbknn. The latter, batch-balanced K-nearest neighbors (Polanski et al., Bioinformatics 2020), is perhaps an obvious target of comparison for mbkmeans (or perhaps not -- mbkmeans does not seem to make explicit claims about the data integration utility of its fast and memory-efficient implementation, which may or may not be relevant). Polanski et al. did not appear in the References section of this paper, and perhaps the authors view it as out of scope, but while scaling up clustering to millions of cells is useful, if the clustering ends up reinforcing artifacts in the provided data, this would seem to reduce the utility. Most users operating at the scale of millions of cells are likely to be integrating datasets, either before clustering, or as part of it, so I feel like this is an issue that needs to be discussed, if not necessarily dealt with.

As our software does not perform any batch correction (only clustering), we do not include specific comparisons to methods and software that perform batch correction as this is out of the scope of the paper. If needed, we assume that any batch correction step occurs prior to clustering (as in the case of BBKNN), though as we note, some normalization techniques like *scran* require an initial rough clustering of the data.

However, we now compare our workflow with one based on BBKNN followed by Leiden clustering. We note that comparing the partition obtained by *mbkmeans* with that defined by the “mouse” variable (Adjusted Rand Index of 0.04) highlights that batch effects do not seem to affect the clustering of the 1.3 million cells dataset. The following text was included in the Results section to describe the comparison between *mbkmeans* and alternative approaches:

“We compared our pipeline with two alternative approaches, based on Louvain and Leiden clustering, implemented in Bioconductor and in the scanpy Python package [41], respectively. Specifically, after obtaining the top 50 PC dimensions, we computed the shared nearest neighbor (SNN) graph and we detected communities in the network via the Louvain algorithm [26]. Alternatively, after normalization and PCA, we performed batch correction using the BBKNN method [42] and Leiden clustering [27] on the resulting network.

Comparing the resulting partitions, we observed that the clustering results of the three methods are qualitatively similar (S15 Fig and S16 Fig) and that both Louvain and Leiden are considerably slower than *mbkmeans*. The Louvain algorithm took 35.5–156 minutes (depending on whether finding an approximate or exact solution) and the Leiden algorithm took 48.5 minutes (S7 Table); this is in contrast to 30 seconds with mbkmeans.”

“We note that the mbkmeans clustering is not driven by batch effects, as shown by the Adjusted Rand Index (ARI) of the cluster labels and the “mouse” variable (ARI=0.04).”

Finally, we have now included the following in the discussion to emphasize the importance of performing batch correction and integration as part of a complete scRNA-seq pipeline:

“This is particularly problematic as experiments now frequently produce millions of cells [51-54], possibly across multiple batches, making it challenging to even load the data into memory and perform downstream analyses including quality control, batch correction and dimensionality reduction.”

The HDF5 clustering benchmark code is useful as a standalone harness, and the authors are to be commended for including it.

We are glad that the reviewer found our benchmark code useful and we thank the reviewer for the positive feedback.

In running the software from its vignette, I found that the necessary library() calls to mbkmeans, TENxPBMCData, scater, and DelayedMatrixStats were omitted from the code snippets. Thus, a user attempting to copy and paste directly from the package vignette will encounter errors. This should be fixed; the package itself is a part of any application note, as one cannot apply a paper directly to a computational problem, and the vignette for a package is an integral part of both its documentation and (to some extent) user testing. Once these missing calls were included, the vignette runs without incident, although the demonstration sample size is so small as to somewhat diminish the utility of the vignette in playing with parameters. From the point of view of a user, the package vignette is vastly more likely to serve as the point of entry than a paper

whose performance evaluations are a bit peripheral to the question of "should I use this method at all, or just use scanpy+bbknn+UMAP+leiden". The latter has some sleight of hand involved (downsampling).

We thank the reviewer for pointing out that the vignette lacked the library() calls, and we have added those in the latest release of Bioconductor (*mbkmeans* version 1.6.1).

The goal of vignettes included with Bioconductor packages is to introduce users to how to use the code in the package ("a document that provides a task-oriented description of package functionality", https://www.bioconductor.org/help/package-vignettes/); traditionally they are not intended to compare with other methods, which is usually done in a research article. Moreover, the vignettes are generally intended to be able to run quickly (they are run at every Bioconductor build), and thus generally use comparatively small datasets. However, we agree that the vignette's use of a small dataset does not appropriately put the purpose of the algorithm (very large datasets) in context, which is confusing for a user. We have updated the vignette to clearly explain that the speed and memory advantages of the technique come in much larger datasets than those used in the vignette. We have also updated the text of the vignette to reference a preprint of this paper (as available on *bioRxiv*) and point users to that manuscript for comparisons with other methods. We also discuss more fully questions of how to choose arguments for large datasets that cannot be held in memory (e.g. batch size), where we summarize the results of this manuscript.

In the above vignette, there does not appear interpretation of why different clustering results are obtained from mini-batch K-means and full K-means (as demonstrated in vignette steps 3.1.2 and 4), nor with k-means++ initialization, especially given the notes in the paper itself. In the paper we find a note that, for batches of k=500 or larger, minibatch k-means recovers the results of classical K-means ("mkbmeans is accurate").

We have added text under that comparison in the vignette to note that *mbkmeans* will not give the same results as *kmeans*, and explain why that is the case along with a reference to the preprint. We also added similar text in the paper to clarify this issue as follows:

> **"Tang and Monteleoni [28] demonstrated that the mini-batch k-means algorithm converges to a local optimum. However, mini-batch k-means follows a different search path than k-means, and therefore does not necessarily converge to the same local optimum as the k-means algorithm."**

The authors note that the employed minibatch k-means method from ClusterR is essentially scaled up and adapted to an HDF5 backend, which (while more challenging than it may appear) is not inherently of broad interest. Thus the demonstration that the package does in fact recover results previously feasible only with much more RAM and CPU seems important.

We thank the reviewer for this comment. We have now compared the performance of *mbkmeans* (data in memory and data stored on disk) to *ClusterR* (only option is data stored in memory). As expected, our HDF5 version of mbkmeans greatly reduces the memory usage whether compared to our in-memory *mbkmeans* implementation or the *ClusterR* implementation (updated Figure S1). Comparing more closely using just our in-memory implementation with that of *ClusterR*, we see that even there, our in-memory *mbkmeans* is more memory-efficient than *ClusterR* for all simulated dataset sizes (75,000 to 750,000 cells), though obviously not with the same order of magnitude as seen with the HDF5 implementation. Furthermore, we also show that *mbkmeans* is as fast as *ClusterR* for similar dataset sizes (Figure S2). Therefore, we have now demonstrated the improvements over *k*-means are not only achieved from the mini-batch *k*-means algorithm itself, but also in the implementation of our *mbkmeans* software package. We have added the following to the manuscript:

> **"In addition, we compared our in-memory *mbkmeans* implementation to the mini-batch *k*-means algorithm implemented in the *ClusterR* R package (S1 Fig). We found *mbkmeans* was more memory-efficient compared to *ClusterR*, demonstrating the improvements over *k*-means are not only achieved from the mini-batch *k*-means algorithm itself, but also in the implementation of our *mbkmeans* software package."**
>
> **"In addition, we found *mbkmeans* performed similarly in computational time compared to *ClusterR*, leading to no loss in performance of speed (S2 Fig)."**

Similarly, hyperparameter tuning is an issue in almost all single-cell RNAseq tasks, especially when it is a primary free parameter (number of centroids) in an algorithm. This seems to deserve somewhat more discussion or automation; the use of the adjusted Rand index (ARI) to gauge fit is useful, so a method to automate the process of evaluation seems germane to the package. Alternatively, implementations of parallel Bayesian optimization exist (e.g. https://cran.r-project.org/web/packages/ParBayesianOptimization/vignettes/tuningHyperparameters.html) and might be appropriate for some of these subtasks.

We agree that picking parameters in clustering routines -- in the case of kmeans the number of clusters -- is always an issue and greatly affects the results. There is a great deal of literature in how to select parameters and addressing this question is beyond the scope of this paper or package. However, we would note that all techniques of choosing the appropriate parameter *k* (or other clustering parameters) require clustering of the data for a range of values. Fast and memory efficient algorithms, like *mbkmeans*, facilitate this process for the user. For example, parallelization of such tasks is only feasible if the base clustering routine has a low-memory usage.

While we do not address how to choose the number of clusters, we do run *mbkmeans* over 16 different values of *k* in our running of a complete pipeline on the full 1.3 million cells (requiring a total of 3 minutes, Table S5) for the purpose of showing the

computational feasibility of evaluating different choices of *k* with *mbkmeans*. Furthermore, we have now added a Section in the package vignette called "Running mbkmeans with multiple values of k" that shows an example on how to run the algorithm for multiple values of k and how to select a reasonable value of *k* using the elbow method.

The authors note that existing clustering methods are memory-intensive and implicitly require loading of entire matrices into memory, whereas mbkmeans does not. The issue of downsampling (property-preserving or otherwise) as a workaround for some of these issues is not really touched upon other than to note that mbkmeans "samples" only the minibatch-sized block of observations required to iteratively employ the method. Whether structure beyond 5-15 centroids is effectively recovered in atlas-sized datasets of the sort that primarily motivate this memory-efficient work is not really discussed in the manuscript that I could see.

We agree that there are widely differing purposes in using small portions of the data in clustering routines. We would separate these into 1) those that ultimately minimize the same objective function, but are algorithmically taking advantage of needing to work on only small portions of the data at the same time. Examples beyond just that of clustering include stochastic gradient descent, MAP Reduce, etc. *mbkmeans* is included in this category. Versus 2) those algorithms that actually change the clustering objective/algorithm. Examples include clustering only a small portion of the data and then assigning the rest of the cells based on those results (as done for instance in the *SC3* Bioconductor package), or repeatedly subsampling the data and creating a consensus clustering on the output (as done in the *clusterExperiment* Bioconductor package). We would agree that some of the methods in this second class really are a work-around for tackling the issue of clustering large datasets, and if the underlying structure is more detailed could miss the finer resolution looked for with large single cell datasets. But the first class of strategies do not impact the ability to minimize the objective function evaluated on all samples and should be considered as simply different algorithm implementations. Specifically, *mbkmeans* is still solving the global optimization problem over all cells (with proof of convergence to a local optimum, like *kmeans*), and should be able to handle different levels of resolution.

We have added text to the introduction to make this clearer:

> **"We would emphasize, however, that while mini-batch k-means only operates on small subsamples of the data at any one time, the algorithm still minimizes the same global objective function evaluated over all samples as in traditional implementations of k-means."**

Furthermore, as mentioned above, we have added a reference clarifying the convergence properties of the mini-batch *k-means* algorithm:

> **“Tang and Monteleoni [28] demonstrated that the mini-batch k-means algorithm converges to a local optimum.”**

I am less interested in "comparing" mbkmeans against itself on a 1.3M-cell dataset than against something like bbknn and/or leiden downstream of scanpy, and I feel that the authors are remiss in not doing so, even if only to contrast the two in terms of platform. The supplementary figures for the bbknn paper are remarkably effective in demonstrating a use case where the popular Harmony method fails to recover biologically relevant similarities (in their use case, the single best studied somatic stem cells in all of biology). Something along these lines seems appropriate in an Application Note, to motivate _why_ a user would apply mbkmeans to their data, instead of simply dumping it to an .h5ad file and ramming it all through scanpy.

We thank the reviewer for this suggestion. We have now expanded the comparison of *mbkmeans* to existing alternatives including the *scanpy* pipeline, which includes the batch-balanced k-nearest neighbors (BBKNN) batch correction step, and two graph-based clustering: Louvain and Leiden, as implemented in R/Bioconductor and Python, respectively. As shown in Supplementary Table S7 of the revised manuscript, *mbkmeans* is considerably faster than both Louvain and Leiden clustering (both using exact and approximate neighbor search). Moreover, Supplementary Figures S15 and S16 of the revised manuscript show that the partitions obtained by the three methods are qualitatively similar.

The following text was included in the Results section to describe these new results:

> **“We compared our pipeline with two alternative approaches, based on Louvain and Leiden clustering, implemented in Bioconductor and in the scanpy Python package [41], respectively. Specifically, after obtaining the top 50 PC dimensions, we computed the shared nearest neighbor (SNN) graph and we detected communities in the network via the Louvain algorithm [26]. Alternatively, after normalization and PCA, we performed batch correction using the BBKNN method [42] and Leiden clustering [27] on the resulting network.**
>
> **Comparing the resulting partitions, we observed that the clustering results of the three methods are qualitatively similar (S15 Fig and S16 Fig) and that both Louvain and Leiden are considerably slower than *mbkmeans*. The Louvain algorithm took 35.5–156 minutes (depending on whether finding an approximate or exact solution) and the Leiden algorithm took 48.5 minutes (S7 Table); this is in contrast to 30 seconds with mbkmeans.”**

Addressing the above issues would strengthen the paper, the software, and the application of the software to users' papers. A fast, memory-efficient implementation of k-means is handy, but if few users apply k-means to cluster single-cell data going forward, then the application note is more of a technical demonstration of issues involved in applying in-memory algorithms to

HDF5-backed out-of-core data. This is not a trivial task, but it's not clear to me whether the audience for it is the same as for an application note.

We thank the reviewer for the feedback. We have now addressed all the above issues. We hope the reviewer will find the updated manuscript compelling for an application note.

Reviewer #3:

The authors developed an R package implementing the min-batch k-means algorithm in a memory-efficient way, enabled application to on-disk data and integrated it with widely used Bioconductor data containers in scRNA-seq analysis. The paper itself is particularly well written, with methods, results and the specific contributions of the work to the field presented clearly.

Their implementation of mbkmeans in an efficient Bioconductor package makes great use of existing computational infrastructure and with full integration in the Bioconductor ecosystem, mbkmeans is immediately useful and useable for people doing single-cell data analyses in R. The example of mbkmeans to provide an initial, fast clustering of cells before normalisation and further downstream analysis steps is a very nice application.

We thank the reviewer for their positive comments and their enthusiasm for our paper.

Overall, we have a small number of comments, mainly with some queries/suggestions where the authors may be able to provide a little more clarity to ease understanding for the reader.

Of course, the authors quite deliberately avoid the lingering question for the reader: "should I use k-means to cluster my single-cell data?" Fair enough for answering that question to fall outside the scope of this paper; nevertheless it would be very useful to know when k-means (esp as implemented in mbkmeans) is a preferable choice for single-cell RNA-seq clustering over popular current graph-based clustering methods like Louvain or Leiden.

We thank the reviewer for this suggestion. We have now expanded the comparison of *mbkmeans* to existing alternatives including the *scanpy* pipeline, which includes the batch-balanced k-nearest neighbors (BBKNN) batch correction step, and two graph-based clustering: Louvain and Leiden, as implemented in R/Bioconductor and Python, respectively. As shown in Supplementary Table S7 of the revised manuscript, *mbkmeans* is considerably faster than both Louvain and Leiden clustering (both using exact and approximate neighbor search). Moreover, Supplementary Figures S15 and S16 of the revised manuscript show that the partitions obtained by the three methods are qualitatively similar.

The following text was included in the Results section to describe these new results:

“We compared our pipeline with two alternative approaches, based on Louvain and Leiden clustering, implemented in Bioconductor and in the scanpy Python package [41], respectively. Specifically, after obtaining the top 50 PC dimensions, we computed the shared nearest neighbor (SNN) graph and we detected communities in the network via the Louvain algorithm [26]. Alternatively, after normalization and PCA, we performed batch correction using the BBKNN method [42] and Leiden clustering [27] on the resulting network.

Comparing the resulting partitions, we observed that the clustering results of the three methods are qualitatively similar (S15 Fig and S16 Fig) and that both Louvain and Leiden are considerably slower than *mbkmeans*. The Louvain algorithm took 35.5–156 minutes (depending on whether finding an approximate or exact solution) and the Leiden algorithm took 48.5 minutes (S7 Table); this is in contrast to 30 seconds with mbkmeans.”

1. Major comments on simulations

1.1 mbkmeans is fast and memory-efficient

It is clear that the original mini-batch kmeans algorithm is more memory efficient compare to the traditional k-means. From the results in Fig1, it is not so clear if the improvement in speed and memory consumption is achieved by the algorithm itself or the implementation of mbkmeans. So it would be helpful (say in a supp figure) to compare mbkmeans to the other R package that implements the same algorithm, for example ClusterR, instead of traditional k-means. It would be preferable to show results from the simulation experiment in this section to speed and memory usage comparison with ClusterR.

In addition to *k*-means, we have now compared the performance of *mbkmeans* (data in memory and data stored on disk) to *ClusterR* (only option is data stored in memory). As expected, our HDF5 version of *mbkmeans* greatly reduces the memory usage whether compared to our in-memory *mbkmeans* implementation or the *ClusterR* implementation (updated Figure S1). Comparing more closely using just our in-memory implementation with that of *ClusterR*, we see that even there, our in-memory *mbkmeans* is more memory-efficient than *ClusterR* for all simulated dataset sizes (75,000 to 750,000 cells), though obviously not with the same order of magnitude as seen with the HDF5 implementation. Furthermore, we also show that *mbkmeans* is as fast as *ClusterR* for similar dataset sizes (Figure S2). Therefore, we have now demonstrated the improvements over *k*-means are not only achieved from the mini-batch *k*-means algorithm itself, but also in the implementation of our *mbkmeans* software package. We have added the following to the manuscript:

“In addition, we compared our in-memory *mbkmeans* implementation to the mini-batch *k*-means algorithm implemented in the *ClusterR* R package (S1 Fig). We found *mbkmeans* was more memory-efficient compared to *ClusterR*,

demonstrating the improvements over *k*-means are not only achieved from the mini-batch *k*-means algorithm itself, but also in the implementation of our *mbkmeans* software package.”

“In addition, we found *mbkmeans* performed similarly in computational time compared to *ClusterR*, leading to no loss in performance of speed (S2 Fig).”

1.2 mbkmeans is accurate

Intuitively, it seems that the ratio between the batch size and the sample size, instead of the absolute value of each, would influence the performance of the algorithm. So it would be nice to have a figure showing the change of ARI and/or WCSS w.r.t the change of the ratio.

While initially the ratio seems to make sense, it actually is quite problematic for the memory performance of the algorithm to use a fixed proportion as a batch size for samples sizes of different orders of magnitude. In particular, memory usage is tied to the absolute batch size, so that a proportional batch size would show continual growth in memory usage and defeat the goal of controlling memory. Of course, if you needed a proportional increase in batch size in order to maintain accuracy, it would be a problem to only use a fixed batch size. However, this is clearly not the case in our simulations, where the accuracy becomes indistinguishable at a very small batch size (500) irregardless of the sample size.

Can the authors provide a practical guide in how to select the batch size w.r.t different sample sizes? Or a suggestion on a lower bound for the ratio where a convergence in performance can be expected? Is the lower bound of 500 suggested in the text completely widely applicable, or are there any further considerations for users to optimise this parameter in practice?

The suggested lower bound of using absolute batch sizes of 500 observations stems from Figure 2 where we compare the accuracy (both ARI and WCSS using simulated data or just WCSS using real scRNA-seq data) of *k*-means and *mbkmeans* (both in memory and on-disk data representations). In our experience, we have found using *mbkmeans* with batch sizes of b=500 or larger lead to no loss in accuracy compared to *k*-means. We found this to be true for different sample sizes (5000, 10000, and 25000). Furthermore, we found that memory-usage and computational speed (elapsed time) were not impacted using batch sizes up to 10,000 cells (Figure 3). Finally, we have added the following text to the manuscript (page 7) as a recommendation for batch sizes for users:

“Therefore, we recommend users to use a batch size of *b* = 10,000, which balances both the results from accuracy (Fig. 2), memory-usage and speed (Fig. 3). Furthermore, since the initialization uses by default the same number of cells as batch size, this gives a robust sample for determining the initial start point.”

This section seems to be evaluating the accuracy of the mini-batch algorithm, does it mean any software implementing this algorithm reaches the same accuracy? Or mbkmeans improved the accuracy compared to ClusterR?

We thank the reviewer for giving us the opportunity to clarify the goal of this section. Our point is not to demonstrate that *mbkmeans* is more accurate than other mini-batch *k*-means implementations. Rather, we aim to show that mini-batch *k*-means, as implemented in the *mbkmeans* package, does not lead to loss of accuracy compared to the full *k*-means. This result is not surprising and confirms results already presented in Sculley (2010).

We have now included the *ClusterR* implementation of mini-batch *k*-means in the accuracy comparison (WCSS), showing no significant differences between the two implementations. More importantly, after 500-1000 cells, there is no difference in accuracy between *k*-means, *mbkmeans*, and *ClusterR*.

We have added the following text to the manuscript to clarify the aim of this analysis:

> **"We note that these results do not depend on our implementation, but on the mini-batch k-means algorithm, as both mbkmeans and the implementation in the ClusterR [21] R package lead to comparable WCSS values (S7 Fig)."**

Does mini-batch k-means guarantee convergence?

Mini-batch k-means has been shown to converge to a local optimum, though it will not necessarily converge to the same local optimum as *k*-means. We have added text in the manuscript to clarify this point:

> **"Tang and Monteleoni [27] demonstrated that the mini-batch k-means algorithm converges to a local optimum. However, mini-batch k-means follows a different search path than k-means, and therefore does not necessarily converge to the same local optimum as the k-means algorithm."**

Are clustering results from different runs on the same data identical?

No, different runs of *mbkmeans* will not be the same since there is randomness in both the selection of the batch as well as the initial starting point. Since the objective function is non-convex, this means that different runs of *mbkmeans* could locate different local minima. We would note, however, that this is not any different than the standard *k*-means algorithm. While *k*-means will always converge to the same solution from the same initialization point (which is not true of *mbkmeans* since there is the additional

randomization of the batch samples), the initialization point for *k*-means is also chosen randomly so that different runs of *k*-means will also have different solutions.

We have added text to that effect,

"Like the k-means algorithm, the mini-batch k-means algorithm will result in different solutions at each run due to the random initialization point and the random samples taken at each point."

1.3 Impact of batch size on speed and memory-usage

Since the highlight of mbkmeans is its memory effectiveness, can the authors provide an analytical expression (or heuristic) for the order of memory usage as a function of the number of clusters k, sample size N and batch size b?

Otherwise, a rough explanation of how the memory usage and running time is likely to change according to the change in k, N and b would also be very helpful for users to manage their expectations. Understandable if the best possible information is the results obtained under various settings in practice.

An rough expression of the memory usage required to store the data and intermediate calculations held in memory at any time for the HDF5 version of mbkmeans is given by $[bG + k(G + b)] * 8 * 1^{-9} GB$, where *G* is the number of genes, *b* is the batch size, *k* is the number of clusters, while that of the in-memory version is $[NG + k(G + b)] * 8 * 1^{-9} GB$, where *N* is the number of samples. We do not have as much insight into the explicit details of the implementation of *k*-means, but based on our simulations, its memory consumption appears to be on the order of $[4NG] * 8 * 1^{-9} GB$, probably due to the Hartigan-Wong implementation which makes several passes and copies of the data at each iteration. These are just rough approximations which focus on the role of the size of the data matrix, *NG*; they match our simulation results in Fig 1, but are not fine-tuned enough to account for the roughly quadratic behavior we see in simulations for changes in the memory usage of the HDF5 versus as a function of *b* (Fig 3) as other parameters are held fixed. These are terms that are clearly a smaller order of magnitude in the memory usage than *NG*, and the discrepancies are likely due to operations beyond the specific amount of data held by our code, such as the underlying code that reads the HDF files or even how memory is handled internally by R. For this reason we do not feel comfortable including these approximations in the text.

However, we provide (via simulation) the change in memory usage as a function of *N* (Fig 1), as a function of *b* (Fig 3), and as a function of *k* (Fig S8), which should allow a user to make judgments for the expected memory usage in their setting.

Vignette

Overall, the vignette is excellent and easily allows a user to get going with mbkmeans. We were able to install the package and run the code in the vignette without any problems. We have the following minor notes:

Missing "library" calls means that the user encounters errors if literally running the code shown in the vignette; we suggest the authors add library() calls for scater, DelayedMatrixStats, TENxPBMCData

We thank the reviewer for pointing out that the vignette lacked the library() calls, and we have added those in the latest release of Bioconductor (*mbkmeans* version 1.6.1).

It would be handy to be able to enter a vector to the 'clusters' argument to compute k-means for various k in one line. Otherwise, a short example in the vignette showing how to neatly run mbkmeans with a range of k values would be helpful, since this is a likely use case for many users.

We have now included a Section in the vignette called "Running mbkmeans with multiple values of k" that shows an example on how to run the algorithm for multiple values of *k* and how to select the "best" *k* using the elbow method.

A walk-through of using on-disk storage would be very handy (or a pointer to resources for the user where they can read up on SCE objects with disk-backed data)

Currently, we recommend the workshop by Peter Hickey titled 'Effectively using the DelayedArray framework to support the analysis of large datasets' given at the Bioconductor 2020 annual conference. This workshop walks through state of the art practices for using SingleCellExperiment objects with disk-backed data. A link to this workshop can be found here:

https://petehaitch.github.io/BioC2020_DelayedArray_workshop/articles/Effectively_using_the_DelayedArray_framework_for_users.html

We have added this link to the package vignette in a new Section called "Working with on-disk data in Bioconductor".

2. Minor comments

2.1 on notation tweaks (Line 57, page 3)

Since Y is a "set" of observations, it should be Y = {y_1, ..., y_N};

This has been corrected.

"into k (\leq N) " could best be k (< N) since it makes no sense if k = N;

This has been corrected.

Please mention ||.|| *denotes L2 norm.*

This has been added.

2.2 Possible typo: in abstract line 3, "analyses" --> "analysis".

We believe “analyses” is correct.
